# Stimuli-activatable nanomedicine meets cancer theranostics

**DOI:** 10.7150/thno.87854

**Published:** 2023-10-02

**Authors:** Haonan Li, Yue Feng, Qiang Luo, Zhiqian Li, Xue Li, Huatian Gan, Zhongwei Gu, Qiyong Gong, Kui Luo

**Affiliations:** 1 Department of Radiology, and Department of Geriatrics, Laboratory of Heart Valve Disease, Huaxi MR Research Center (HMRRC), Laboratory of Stem Cell Biology, National Clinical Research Center for Geriatrics, Frontiers Science Center for Disease-Related Molecular Network, State Key Laboratory of Biotherapy, West China Hospital, Sichuan University, No. 37 Guoxue Alley, Chengdu 610041, China.; 2 Functional and Molecular Imaging Key Laboratory of Sichuan Province and Research Unit of Psychoradiology, Chinese Academy of Medical Sciences, Chengdu 610041, China; 3 Department of Radiology, West China Xiamen Hospital of Sichuan University, 699 Jinyuan Xi Road, Jimei District, 361021 Xiamen, Fujian, China.

**Keywords:** exogenous stimuli, Endogenous stimuli, stimuli activation, nanomedicine, cancer theranostics

## Abstract

Stimuli-activatable strategies prevail in the design of nanomedicine for cancer theranostics. Upon exposure to endogenous/exogenous stimuli, the stimuli-activatable nanomedicine could be self-assembled, disassembled, or functionally activated to improve its biosafety and diagnostic/therapeutic potency. A myriad of tumor-specific features, including a low pH, a high redox level, and overexpressed enzymes, along with exogenous physical stimulation sources (light, ultrasound, magnet, and radiation) have been considered for the design of stimuli-activatable nano-medicinal products. Recently, novel stimuli sources have been explored and elegant designs emerged for stimuli-activatable nanomedicine. In addition, multi-functional theranostic nanomedicine has been employed for imaging-guided or image-assisted antitumor therapy. In this review, we rationalize the development of theranostic nanomedicine for clinical pressing needs. Stimuli-activatable self-assembly, disassembly or functional activation approaches for developing theranostic nanomedicine to realize a better diagnostic/therapeutic efficacy are elaborated and state-of-the-art advances in their structural designs are detailed. A reflection, clinical status, and future perspectives in the stimuli-activatable nanomedicine are provided.

## 1. Introduction

Cancer theranostics (e.g., chemotheranostics, radiotheransotics, immunotheranostics, and phototheransotics) have shown tremendous promise in cancer management 1. A nanoscale size or a nanostructure endows these theranostic products with a high specific surface area, tunable physiochemical properties, flexible imaging/therapeutic functions, and improved biocompatibility. Theranostic nanomedicine has been designed and constructed for better cancer management than current theranostic products for diagnosis and therapy 2. For example, disease-specific biomarker-targeting ligands have been incorporated into nanostructures with imaging properties for early cancer detection and accurate profiling of tumors. Furthermore, visualization of chemotherapeutic drugs, photo-/radio-/sono-sensitizers, tumor vaccines, immune regulators, and immune cells via nanostructures with imaging properties could revolutionize the entire cancer management procedure 3, 4. Additionally, versatile modification and/or manipulation of nanostructures create greater room for cancer theranostics, for example, real-time single/dual/multiplex imaging modality-guided or imaging-assisted cancer treatment 5, 6.

Theranostic nanomedicine can reach tumor sites with a high cargo load through the enhanced permeability and retention (EPR) effect or the modification with active targeting moieties 7, while controlled or smart release of the cargo in the nanomedicine can avoid systemic adverse effects and circumvent a compromised potency due to burst or pre-leaked release. In this context, stimuli-activatable tactics in designing and constructing theranostic nanomedicine have been proposed. Specifically, unique characteristics of the tumor microenvironment (TME), including a low pH, a high redox level, hypoxia, and overexpressed specific enzymes, have been explored for stimuli-activatable theranostics to achieve tumor-specific management 8. Moreover, mechanical forces exerted from light, radiation, magnetic field, and ultrasound have been employed for vascular transportation, activation, or degradation of theranostic nanomedicine to realize potent, targeted therapy with remarkably reduced adverse effects. These bioactivatable nanomedicinal products are often designed with an activatable structure before reaching tumor sites, while their structures are disassembled, re-assembled, or activated in response to the TME characteristics or external physical forces 9, reaching a balance between their on-target efficacies and off-target toxicity for better cancer management.

After these exogenous/endogenous stimuli alter the structure of the nanomedicine products, imaging agents/drugs could be released from the nanostructures for directly exerting imaging/therapeutic effects or they are aggregated for enhancing their imaging and/or therapeutic potencies. In terms of imaging, specific endogenous stimuli or exogenous physical forces could help expose, activate, or aggregate the imaging moiety in the nanomedicine, thus boosting imaging signal intensity. Novel strategies for stimuli-triggered signal-switch in the nanomedicine have emerged, for instance, manipulating the relative distance between a signal emitter/enhancer and a quencher in a hollow nanostructure. The nanostructure could be loaded with imaging, therapeutic, or theranostic agents and it is also equipped with modifiable gatekeepers or degradable compartments that are sensitive to a specific stimulus. In response to the stimulus, the nanostructure or the linker could be deformed/degraded to increase the distance between the pro-quenched or protective agents and their signal emitters/enhancers or bioactive agents, thus triggering a “switch-on” for its activatable or switchable imaging function 10. Furthermore, since some of inorganic nanoagents have imaging/therapeutic function, the detachment of their surface coating may lead to aggregation of inorganic nanoagents for enhanced imaging (e.g*.*, computed tomography (CT), T2-weighted image (T2WI) of magnetic resonance imaging (MRI), photoacoustic imaging (PAI), and aggregation-induced emission (AIE)) and/or antitumor therapeutic efficacies (e.g*.*, photothermal therapy (PTT) and radiotherapy (RT)). In addition, to design stimuli-activatable theranostic nanostructures, theranostic nanoagents (e.g*.*, MnO_2_ and polydopamine) with inherent stimuli-responsive imaging/therapy properties could be utilized to boost the therapeutic/imaging efficacy. A few stimuli-activatable theranostic strategies, including simultaneous/sequential/cascade activation, integration of dual/multiplex endogenous stimuli, and combination of endogenous stimuli with exogenous ones have gained popularity.

**Figure 1:**
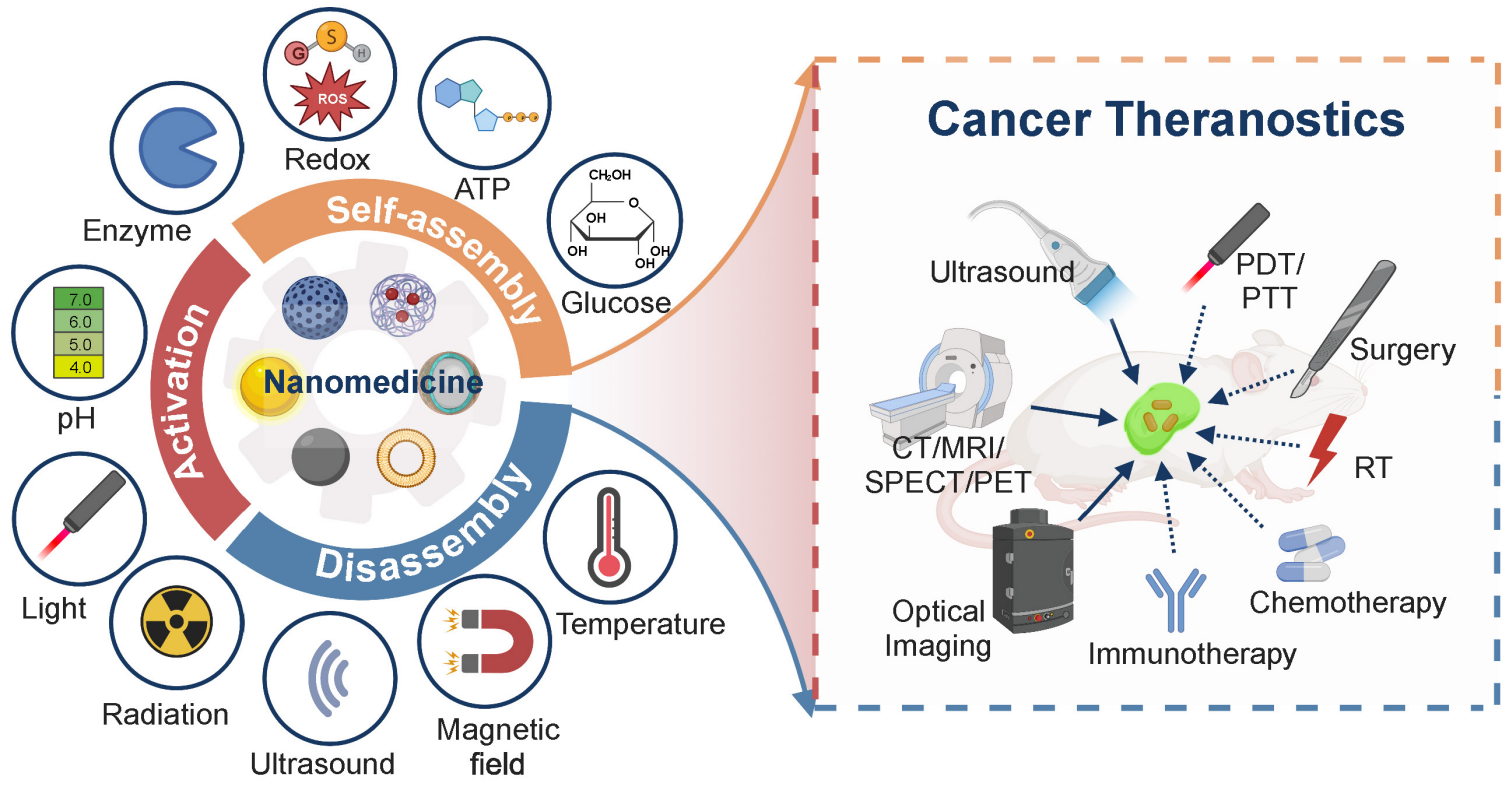
Scheme for stimuli-activatable nanomedicine-assisted cancer theranostics. Activatable cancer nanomedicine is designed to be sensitive to endogenous (a low pH, over-expressed enzymes, an elevated concentration of active small molecules including redox agents, ATP, and glucose) and/or exogenous stimuli (light, radiation, ultrasound, magnetic field, and temperature), thus improving the efficacy of cancer theranostics, an integration platform of imaging modalities indicated by solid arrows (ultrasound, CT, MRI, PET, SPECT, and optical imaging) and therapeutic approaches indicated by dashed arrows (PDT/PTT, surgery, RT, chemotherapy, and immunotherapy). PET: positron emission tomography; SPECT: single photon emission computed tomography; PDT: photodynamic therapy; RT: radiotherapy.

A few stimuli-activatable nanostructures have entered clinical trials or used in clinical practice, such as thermo-sensitive DOX-loading liposomes (ThermoDox®) and ONM-100 (a pH-sensitive fluorescent polymeric nanoparticle conjugated with indocyanine green). They have exhibited improved bioavailability and/or an encouraging diagnostic efficiency 11. These inspiring results indicate that the application of stimuli-activatable tactics in developing theranostic nanomedicine could hold great potential for treating advanced cancer. In this review, we reveal the indispensable role of theranostics in the long-lasting clinical need for cancer management. Stimuli-activatable strategies for designing nanomedicine for theranostics are elaborated, with emphasis on endogenous/exogenous stimuli and their corresponding activatable ligands/structures, as well as the activatable mechanisms, which have not been systematically reviewed before. Additionally, state-of-the-art application of activatable cancer theranostic nanomedicine is surveyed. Finally, the current clinical status, along with a reflection and future perspectives of activatable theranostic nanomedicine are presented (**Figure 1**).

## 2. Stimuli-activatable strategies for nanomedicine-assisted cancer theranostics

### 2.1. Nanomedicine embraces cancer theranostics

**Figure 2:**
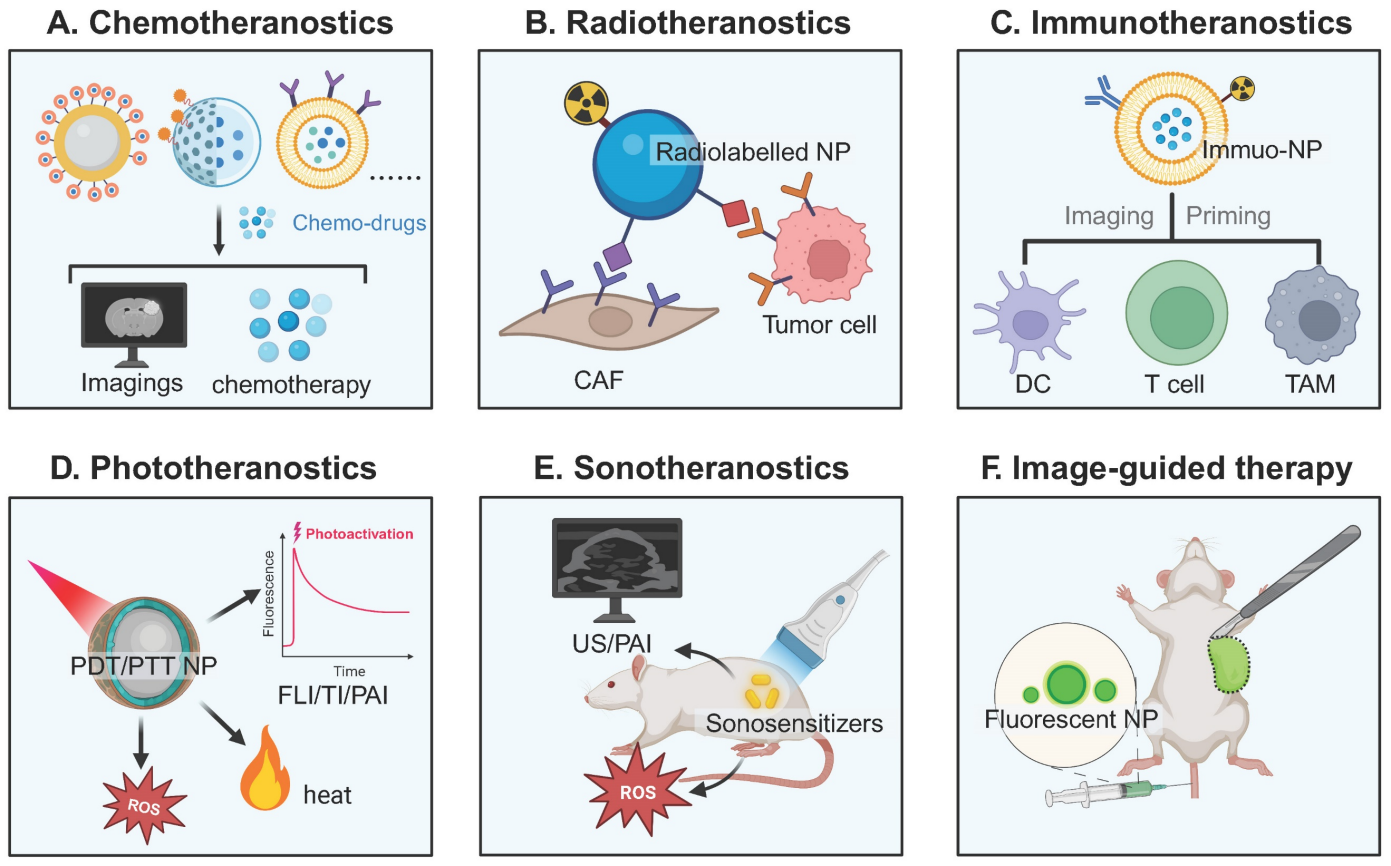
Schematic of nanomedicine in different theranostic regimes based on treatment approaches. A) Chemotheranostics: various nanoformulations deliver chemotherapeutic molecules (some of them are optically visible) and imaging agents. B) Radiotheranostics: diagnostic/therapeutic radionuclides-labeled nanoparticles target tumor cells or CAFs. C) Immunotheranostics: nanomedicine is designed to image and prime immune cells, including DCs, T cells, and TAMs. D) Phototheranostics: light induces response from nanomedicine to generate imaging signals in FLI/TI/PAI or exert therapeutic effects of therapeutic agents (ROS and heat) in fluorescence imaging-guided PDT and/or PAI-guided PTT. E) Sonotheranositcs: ultrasound at a low intensity triggers sonosensitizers to improve ultrasound imaging contrast and/or generate toxic ROS in tumor cells. F) Image-guided therapy including image-guided surgery and image-guided cell therapies: pre-injection of fluorescent NPs or other optically visible probes aids in delineating tumor margins or sentinel lymph nodes for surgery. NP: nanoparticle; CAF: cancer-associated fibroblast; DC: dendritic cell; TAM: tumor-associated macrophage; FLI: fluorescence imaging; TI: thermal imaging; ROS: reactive oxygen species; US: ultrasound; PAI: photoacoustic imaging.

With tunable imaging properties and improved pharmacokinetics, nanomedicine emerges as an important addition to the existing imaging and therapeutic modalities 12. The nanomedicine for cancer theranostics works in various regimes based on therapeutic modalities, including chemotheranostics, radiotheranostics, immunotheranostics, phototheranostics, sonotheranostics, and image-guided therapy (**Figure 2**).

Generally, a variety of theranostic nanoplatforms, including supramolecular architectures, polymeric architectures, inorganic architectures, and hybrid architectures have emerged to hold one single agent or both imaging and therapeutic agents for synergistic theranostics 13, 14. The intratumoral or intranodal distribution of visible nanoparticles have the potential of quantitatively assessing their tumor accumulation/tumor penetration/cellular internalization/lymph node infiltration, spatiotemporally monitoring drug release, accurately predicting therapeutic response, and enabling effective image-guided therapy.

### 2.2. Stimuli-activatable strategies: why adopt them?

There lie several obstacles in the application of nanomedicine-assisted cancer theranostics, including high background noises for imaging, long-term body retention, and unspecific/uncontrolled drug release 15. To overcome these obstacles, a few stimuli-activatable strategies have been proposed for developing cancer theranostic nanomedicine in response to various unique TME characteristics and exogenous physical stimuli. Response of the nanomedicine to these stimuli results in stability/shape/charge/size alterations and prompts self-assembly, disassembly, or activatable function transformation, eventually contributing to an improved theranostic performance. Specifically, these stimuli-activatable strategies have the following advantages.

i. Enhanced tumoral biodistribution, penetration, and retention of activatable theranostic nanomedicine. Endogenous stimuli can break the linker of the nanomedicine to detach protective coatings such as PEG or DNA threads 16, resulting in a decrease in the nanoparticle size, in-situ formation of a nanoassembly, negative-to-positive charge reversal, or release of active agents. Meanwhile, exogenous stimuli can convert an inactive status of theranostic agents (e.g*.*, PDT/PTT nanoagents and sonosensitizers) into active one. Besides, they can remotely alter the TME (e.g., improving vascular penetration) and the *in vivo* behavior of the nanocarrier, leading to improved tumor accumulation and controllable release kinetics 17. Through exerting endogenous or exogenous stimuli on the nanomedicine, homogenous intratumoral distribution, enhanced tumoral penetration, and prolonged tumoral retention of imaging/therapeutic agents can be achieved via transformation of the nanostructure of the nanomedicine including self-assembly, disassembly, or activation 18, 19. For example, stimuli-triggered size reduction of nanomedicine with 100-200 nm (an optimal size for the EPR effect) to 7-50 nm facilitates deep penetration in the solid tumor tissue, while a reduced size < 7 nm enables internalization nuclear targeting 20.

ii. Activatable imaging. The stimuli-activated strategy for designing the nanomedicine for imaging, especially Near-Infrared-II fluorescence imaging and photoacoustic imaging, could remarkably improve the signal-to-noise ratio (SNR) with reduced background noises and enable imaging at a deep distance compared with conventional probes with an “always-on” mode 21. Notably, the distance between a fluorescent emitter and a quencher or a T1/T2 MRI probe can be manipulated for an off-to-on switch to improve the imaging performance 22. For example, a low pH, a high ROS level, a high glutathione (GSH) concentration, or an electric field could help detach surface coatings and extend the distance between Fe_3_O_4_ and Gd/Mn, leading to a switch-on of the T1 signal 23-25. Ratiometric optical imaging of different cellular locations after single or dual light excitation can also be realized by utilizing the distinctive distribution of certain stimuli 26. Meanwhile, stimuli-induced aggregation of imaging nanoagents is conducive to contrast intensification in CT, PAI, and PET/SPECT. In addition, optical nanoprobes, PAI nanoagents, quenched contrast agents, afterglow, and luminescence nanomedicine can be activated by exogenous stimuli (light and X-ray). Activatable imaging of elevated endogenous stimuli generated by therapy, such as ROS and caspase-3, can be employed for therapeutic response assessment via using corresponding stimuli-activatable imaging nanoagent 23, 27.

iii. Biodegradability. After the initial imaging evaluation or therapy, these stimuli-activatable nanostructures have released their cargos, and their nanocarriers are ready for clearance from the body 28, 29, particularly the inorganic one, since long-term retention of these nanocarriers in the human body could result in undesirable toxicity. For instance, stimuli-activated size shrinkage of the nanostructures into renal-clearable nanoassemblies (size < 5.5 nm) aids in their fast elimination 30. Moreover, the addition of stimuli-sensitive linkers to the polymer backbone facilitates their degradation into small fragments (Mw < 45 kDa) for renal excretion after cleavage of these linkers.

iv. Reduced toxicity. Surface shielding of the nanomedicine via stimuli-activatable ligands (e.g., protease-cleavage substrates) is a masking method to alleviate severe side effects of the imaging/therapeutic agents in the nanomedicine in adjacent normal tissues and enable on-demand release of them in tumor tissues.

**Figure 3:**
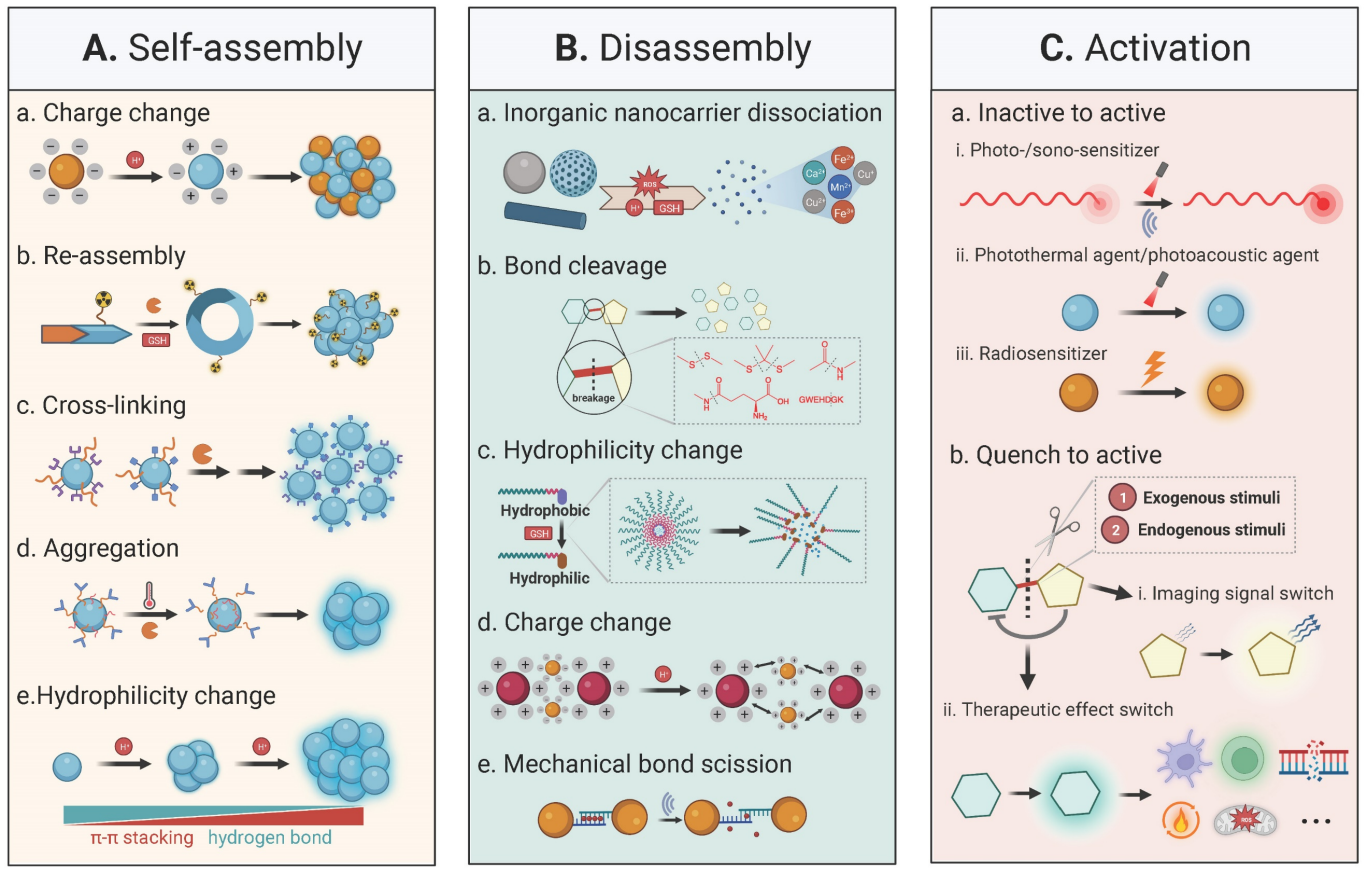
Illustration of self-assembly, disassembly, and activation of nanomedicine. A) Interactions between molecular complexes or nanoparticles, including hydrophobic interaction, hydrogen bonding, electrostatic interaction, host-guest interaction, and dipole-dipole interaction, as well as magnetic or electric forces contribute to stimuli-induced self-assembly. B) Disassembly is realized through morphological/structural transformation/dissociation, bond cleavage, removal of hydrophilic units, charge change, mechanical bond scission, deprotection, cascade reaction, and fragmentation to monomers or oligomers. C) Introducing an activatable energy source or removing a quenched chemical moiety induces the activation of functional moieties in the nanomedicine.

Endogenous stimuli are often considered for stimuli-activatable nanomedicine prodrugs in current pre-clinical practices, while exogenous stimuli are explored for synergistically combined treatment. Response to either endogenous or exogenous stimuli results in in-situ self-assembly, disassembly, and functional activation in the stimuli-activatable nanomedicine; self-assembly and disassembly represent two typical types of structural transformation during the procedure of stimuli-activation, and activation refers to different activation modes of specific function of the nanomedicine (**Figure 3**).

Overall, the ultimate goal of cancer nanomedicine is to achieve the maximum therapeutic benefit with tolerant toxicity, thus stimuli-activatable strategies are the most preferable option to fabricate the nanomedicine.

### 2.3. Endogenous or exogenous stimuli and their corresponding activatable ligands

With joint efforts by cancer biologists, biomedical scientists, chemists, and biomaterial scientists, unique features of the TME have been unveiled and chemical ligands or peptides that are sensitive to these features have been thereafter developed. A brief introduction of various identified endogenous/exogenous stimuli is presented below.

**Low pH.** Tumor cells are conventionally featured with a pH gradient inside cells and between cells: intracellular (pH_i_ ≥ 7.2) except for early endosomes (pH 6.3), late endosomes (pH 5.5), and lysosomes (pH 4.7) and extracellular (pH_e_ = 6.7-7.1) 31. Cancer nanomedicine often experiences the pH gradient during the transportation process from a physiological pH of 7.4, an extracellular pH of 6.7-7.1, and an acidic endosomal/lysosomal pH of 4.7-6.3.

**ROS.** Reactive oxygen species (ROS) are mainly composed of superoxide (O_2_^•-^), hydroxyl radical (•OH), nitric oxide (NO•), hydrogen peroxide (H_2_O_2_), singlet oxygen (^1^O_2_), and organic hydroperoxides (ROOH) 32, 33. The main sources for endogenous ROS are mitochondrial metabolism, peroxisomes, and the activity of the transmembrane NADPH oxidases family 34, 35. In addition, exogenous mechanical forces (e.g., light and radiation) can disrupt the redox imbalance, leading to an elevated ROS level.

**GSH.** Glutathione (GSH), a low-molecular-weight peptide, consists of glutamate, cysteine, and glycine. There is a three-order-of-magnitude difference in the GSH concentration in tumor cells, *i.e.*, 2-10 μΜ in extracellular spaces and 1-10 mM in intratumor cells 36.

**Over-expressed enzymes.** Tumor-associated enzymes are over-expressed in different subcellular localizations. These over-expressed enzymes with their locations are detailed: extracellular environment (matrix metalloproteinase) 37; cell membrane (alkaline phosphatase, aminopeptidase N, and γ-glutamyltranspeptidase) 38; cytosol (transglutaminase, autophagy-related 4B cysteine peptidase, nitroreductase, and caspase-3/7) 39; lysosome (cathepsin B, β-galactosidase, and β-glucuronidase) 40, 41; endoplasmic reticulum (carboxylesterase and protein tyrosine phosphatase 1B) 42; Golgi (furin) 43; mitochondrion (enterokinase) 44; and nuclear (endonuclease histone deacetylases) 45.

**Elevated ATP levels.** The adenosine-5'-triphosphate (ATP) level in an intracellular environment (1-10 mM) of tumor cells is much higher than that in an extracellular environment (<0.4 mM) 46.

**Elevated glucose metabolism.** Accelerated aerobic glycolysis or a rapid cellular glucose metabolic rate to support tumor cell proliferation, which is often accompanied with a high glucose flux, is recognized as a hallmark of cancer 47. Glucose oxidase (GOx), an enzyme which is sensitive to intracellular glucose, has been widely exploited to prepare glucose-responsive nanomedicine for cancer theranostics 48.

**Hypoxia.** Tumor hypoxia originates from vigorous tumor metabolic activities and a deficiency in the O_2_ transfer from vascularity at a long distance away. Euhypoxia (a physicochemical gradient of oxygen), nitroreductase, and their associated features (adenosine, acidosis, and nutrient deficiencies) are quite distinctive in the TME 49.

**Other endogenous stimuli.** Other abundantly-expressed agents in tumor areas, such as H_2_S 50, nitric oxide (NO) 51, metal ions (Ca^2+^) 52, and TK1 mRNA 53, have also been exploited as endogenous stimuli for activatable nanomedicine.

**Light.** Light is used as an activation energy source for nanomedicine-assisted multiplexed therapy (PDT, PTT, and surgery) and imaging (PAI, fluorescence imaging, and thermal imaging) modalities. Without exposure to light, the activatable nanomedicine remains dormant and inefficacious. Upon exposure to light, these responsive agents are activated to realize generation of toxic agents (ROS and heat), stimuli-triggered drug release, and a turn-on of imaging 54, 55.

**Ultrasound.** Ultrasound, a widely used imaging tool, can directly trigger the tumor-killing effect via ultrasonic cavitation and a mechanical and thermal effect in the form of high-intensity focused ultrasound (HIFU), as well as indirectly induce the opening of the blood-brain barrier, destruct imaging-guided gas-filled microbubbles to exert physical stress to cancer cells, and generate sonoluminescence for cancer detection 56.

**Radiation.** Radiation (e.g., X-ray, γ-ray, other charged particles) with an intensive energy can directly induce the degradation of prodrugs and inorganic nanostructures, such as sulfonyl azide- and phenyl azide-caged prodrugs of pazopanib and doxorubicin 57 and a hierarchical metallic supra-nanostructure with thin Au branches connected with Ag nano-linkers 58. Furthermore, radiation can indirectly generate ROS or hydrated electrons in tumors, resulting in the breakage of ROS-activatable linkers or quaternary ammonium masking groups 59. Notably, the unlimited penetration capacity of radiation allows for enhancing photodynamic therapy, fluorescence imaging, and X-ray-excited persistent luminescence for deeply-seated tumors 60.

**Magnetic field.** Magnetic nanoparticles or nanorobots, particularly, the Fe-based one, can be driven by an external magnetic field (e.g., an alternating magnetic field (AMF)) to achieve their localized accumulation and/or generate magnetic hyperthermia. In this procedure, magnetic particle-assisted imaging or T2WI MRI as well as the magnetothermal effect-triggered cargo release from the nanomedicine can be realized 61, 62.

**Thermal**. The localized thermal effect can be elicited by focused ultrasound, magnetic hyperthermia, light-activated photothermal nanoagents, microwave thermal therapy, and microwave dynamic therapy 63. The rise in the local temperature can help generate thermal images, boost the responsiveness of temperature-sensitive linkers, activate thermally activated fluorescent (TADF) nanoagents, and improve the activity of therapeutic enzymes 64.

The endogenous/exogenous stimuli-activatable linkers and their activatable reaction mechanisms have been well summarized elsewhere 9, 65. Herein, we list representative linkers and their chemical structures (**Table 1**). To note, biodegradable inorganic nanostructures, such as mesoporous organosilica and metal oxide (e.g., CaCO_3_ and MnO_2_), are a major type of stimuli-activatable nanomedicine and an important supplement to this Table 66, 67.

Efforts have been devoted to exploring rational design and elegant manipulation of activatable linkers in the nanomedicine, as well as developing efficient alternative ones. An ultra-pH-sensitive nanoparticle library with precisely tuned pH transitions (pHt = 4.0-7.4) was prepared by co-polymerization of a tertiary amine-containing monomer with a group of non-ionizable monomers, and the hydrophobicity of the non-ionizable monomer played an important role in the pH tunability 94. Moreover, chemical structures that are sensitive to dual or multiple stimuli have attracted great attention. For example, poly (*N,N*-dimethylaminoethyl methacrylate) (PDMAEMA) can be both pH-activatable and thermo-activatable 95. Several redox-activatable bonds with dual ROS/GSH-responsivity were designed on basis of sulfur-tellurium-sulfur and sulfur-selenium-sulfur hybrid chalcogen bonds 96. A generalized polymeric delivery system by integrating various stimuli-activatable bonds via logic gates has been proposed for controlled sequential release of drug cargos 97. Besides, the release/activation modes, such as immediately activated, sustainedly activated, progressively activated, and pulsatively activated, could be explored for optimized design of various nanostructures for the activatable nanomedicine.

Taken together, stimuli-enabled nanostructural transformation through stimuli-activatable linkers in the nanomedicine has been reported to improve the biodistribution of a variety of drugs, such as chemotherapeutic agents, immuno-cytokines, and PROteolysis Targeting Chimeras. Concerns over the sensitivity of activatable bonds to other stimuli have been raised when the nanomedicine is prepared, thus novel synthesis methods, such as supercritical fluid-assisted fabrication, have emerged 98. Activation/cleavage of endogenous stimuli-activatable linkers/chemical groups often consume these endogenous stimuli (ROS, GSH, and protons); in this procedure, the adverse effects induced by these stimuli, such as promoting tumor progression and retaining an immunosuppressive TME, may be alleviated. However, this activation process does not consume enzymes. It may be hypothesized that the inactivation of an enzyme through enzyme-responsive linkers could help delay tumor progression or achieve a better prognosis after the breakage of the enzyme-activatable linkers in the nanomedicine.

## 3. State-of-the-art advances in stimuli-activatable nanomedicine for cancer theranostics

### 3.1. Cancer theranostics and theranostic nanoagents

In the current clinical setting, cancer theranostics refer to imaging-guided therapy and radiotheranostics using diagnostic/therapeutic radionuclides pairs. The former is the therapeutic intervention with the guidance of real-time imaging, such as ultrasound-guided punctuation and resection, tumor removal based on stained fluorescent images, CT/MRI-guided delineation of the tumor location for precise radiotherapy, and gastroscopy or proctoscopy-guided surgery. With the application of nanotechnology in cancer theranostics, theranostic nanoagents by incorporating an agent with simultaneous imaging and therapy properties or multiple diagnostic/therapeutic agents into one single nanocarrier 99 have been developed for improved imaging-guided therapy, quantitative control of the released drugs, and extended theranostic regimes (**Table 2**).

### 3.2. Stimuli-activatable nanomedicine in theranostic application

In the context of stimuli-activatable cancer theranostics, endogenous/exogenous stimuli have been explored for developing stimuli-responsible nanomedicine to improve its therapeutic outcome accompanied with a bio-tolerable profile (**Table 3**).

#### 3.2.1. pH-activatable theranostic nanomedicine

Tumor acidosis with a low pH has been widely used as an endogenous trigger to break pH-cleavable bonds and pH-sensitive biodegradable materials for activating pro-theranostic agents. Specifically, coordination bonds that are cleaved by protonation include carboxyl, imidazolyl, amino, amide, pyridine, phenolic hydroxyl, daunosamie, vinyl-ether, and vinyl-amino 126, 127. And the general pH-sensitive nanocarriers can be divided into three main types: inorganic (e.g., calcium phosphate, MnO_2_, CaCO_3_), organic (e.g., polydopamine and polyaniline), and hybrid (e.g., zeolitic imidazolate framework) 128. Generally, surface charge transformation from negative or neutral to positive, or detachment of protective/targeting coating layers aids in improving tumoral penetration and accumulation of the pH-sensitive nanomedicine 129, 130. It is noted that the acidotic transistor nanomedicine with a pH-sensitive proton transistor can amplify and convert a subtle pH change into sharp membranolytic activities, eliciting a robust tumor-killing effect 131.

**Figure 4:**
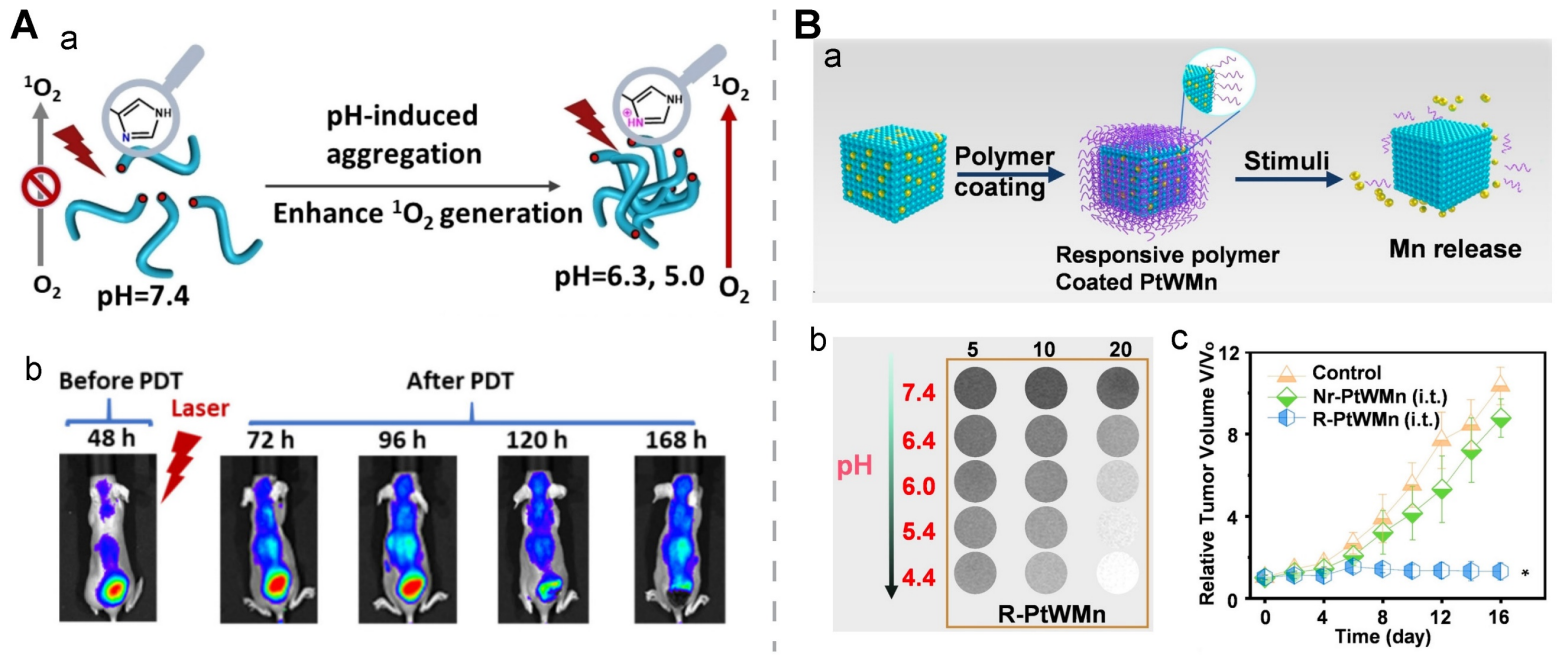
pH-activatable cancer theranostics. A) pH-induced aggregation of a porphyrin-peptide-based nanofiber via protonation for PDT and fluorescent imaging-aided prognosis. a) Schematic illustration. b) Fluorescence images of the mice at various time points post-injection. Reproduced with permission 132. Copyright 2022 the Authors, published by Wiley-VCH. B) An activatable polymer-coated ternary alloy for MRI and ferroptosis therapy. a) Schematic design to illustrate that the detachment of activatable polymer coatings could promote release of metal ions in the core of the nanomedicine. b) T1-weighted MR images of R-PtWMn with different concentrations at different pH values. c) Tumor growth curves after the treatment with the polymer-coated alloy and controls. Reproduced with permission 133. Copyright 2022, Wiley-VCH.

**Organic nanomedicine.** Tumor acidosis-induced aggregation of a nano-assembly could enhance its imaging/therapy performance. For example, a PEGylated porphyrin-peptide-based nanofiber was developed with a negative ζ-potential. It was experimentally confirmed that the imidazole group in the nanomedicine would be protonated in an acidic microenvironment, contributing to an aggregation status by reducing their electrostatic repulsion. The pH-activatable aggregation resulted in strong fluorescence intensity in the tumor for up to 9 days post-treatment and potent ^1^O_2_ generation for effective PDT. This long-term imaging ability was favorable for prognostic monitoring (**Figure 4A**) 132.

pH-induced disassembly of nanomedicinal structures is readily accomplished in the TME and this strategy is the most widely used for developing pH-activatable nanomedicine. A hollow metallosupramolecular nanocapsule, constructed from the polymerization of boronic monomers and catechol monomers, the incorporation of Fe^III^ via the Kirkendall effect, and the loading of DOX, was applied to realize a cascade pH-activatable PAI-guided photothermal-chemotherapy. Initial tumor acidosis-induced “negative to positive” charge transformation was achieved by a combined effect of reversible phenolic hydroxyl ionization and catechol-Fe^III^ coordination; as the pH value of the buffer changed from 7.4 to 7.0 and then 6.5, this activatable nanocapsule showed a steady increase in the ζ-potential but a negligible change in the size distribution. A second wave of C=N cleavage in the endosome (pH < 5.5) enabled robust release of the encapsulated DOX. The PA intensity reached a peak at 6 h post-injection of this nanocapsule in the MCF-7 breast tumor-bearing mice, and this time point was determined to be optimal for the NIR treatment 134.

** Inorganic nanomedicine.** pH-induced structural transformation of inorganic nano-assemblies with surface coating modifications (e.g., a mixed-charge zwitterionic surface) contributes to enhanced performances of imaging (T2WI, CT, PAI) and therapy (PTT, PDT, and RT). pH-activatable structural collapse of inorganic nanoparticles, such as the expansion of pores in mesoporous silica, has been found to promote on-demand sustainable release of drugs and bioactive metal ions, facilitate deep cargo penetration in the tumor tissue, and reduce long-term retention of inorganic nanostructures 135. In addition, an acidic lysosomal environment in tumor cells was found to facilitate the release of cargos (organoselenium SED-1b and SPIONs) from PLZ4 ligand-modified PLGA nanoparticles (PLZ4@SeD); the SED-1b release rate increased from ~30% to around 80% at 72 h when a pH 5.3 buffer was used. The *in vivo* MR performance was examined in three fresh intact bladders resected from bladder cancer patients in the manner of intravesical instillation of PLZ4@SeD. A time-dependent and tumor-specific slight T2WI enhancement was observed. As demonstrated in the bubble-formation tests and MTT assays, PLZ4@SeD imposed a strong cytotoxicity effect on a few cancer cell lines (EJ, J82, T921, SV-HUC-1, MCF7, and HepG2) in a normoxic environment and exhibited a strong oxygen-generation ability via the Fenton reaction. Moreover, the antitumor therapeutic effect of PLZ4@SeD suggested that it could be a potent ROS nanogenerator and a hypoxia reducer, which were confirmed with EJ cells in various tests (cell migration, cell cycle arrest, apoptosis, and ROS generation) 136. In another study, a low pH in the lysosomes promoted rapid disintegration of ultrasmall γ-Fe_2_O_3_ and release of Fe^3+^ ions/DOX from UNA-γ-Fe_2_O_3_@PP@Dox@PF with an excellent photothermal conversion efficiency (PCE) of 59.85%. This pH-activated disassembly behavior improved the imaging performance with a dynamic T2-T1 MRI change; under a 7.0 T MR scanner, the *in vitro* r_2_ and r_1_ value of this nanostructure was 146.23 mM^-1^s^-1^ and 0.2 mM^-1^s^-1^ at pH 7.4, respectively, however, the r_2_ dropped to 41.3 mM^-1^s^-1^ and the r_1_ rose to 0.39 mM^-1^s^-1^ at pH 5.5. The anti-tumor effect was confirmed by the synergistic action from the released DOX, a rise in the localized temperature, and the released Fe^3+^ ions. Conversion of Fe^3+^ to Fe^2+^ was accompanied by the generation of •OH under GSH and H_2_O_2_, leading to chemodynamic therapy, mitochondria damage, and an increase in lipid peroxidation. DOX-induced DNA damage and thermal damage produced by NIR-irradiated UNA-γ-Fe_2_O_3_ exerted additive tumor-killing effects, contributing to a synergistic antitumor therapy with a tumor inhibition rate of 98.6% 137.

**Organic-inorganic hybrid nanomedicine.** Organic coatings often serve as a protective and/or stimuli-activatable layer for an inorganic core in the organic-inorganic hybrid nanomedicine. For example, a pH-activatable polymer was employed as a gatekeeper for reserving manganese (Mn) ions in a ternary PtWMn alloy via doping multivalent Mn ions (Mn^2+^ and Mn^3+^) to a Pt or W nano-cubic skeleton, to obtain the R-PtWMn nanomedicine. The pH-activatable polymer was protonated in an acidic tumor microenvironment and it underwent a hydrophobic-to-hydrophilic transition. Upon exposure to the stimulus *in vitro*, pH-dependent •OH generation, GSH consumption, oxygen generation, and a high level of ferroptosis were found for R-PtWMn, while negligible changes for Nr-PtWMn, a non-responsive control nanomedicine. Moreover, the released Mn ions from the activatable nanostructure contributed to a rise in the pH-dependent r_1_ and r_2_ values at 7.0 T MRI, as well as an enhanced imaging contrast in the tumor sites of the 4T1-bearing mice at the first six hours post-injection. The tumor inhibition rate of the R-PtWMn-treated group exceeded that of the Nr-PtWMn-treated group as well as the groups treated with sham (*i.t.*) and sham (*i.v.*). This study suggested that this organic-inorganic hybrid nanomedicine could realize ferroptosis through real-time MR monitoring in a tumor acidosis-activatable manner (**Figure 4B**) 133. In another study, partially-released zinc ions from the core in an inorganic nanostructure (microporous ZIF-8 NPs) in response to the tumor acidosis were employed to tune the hydrophilicity and surface charge of a sulfoxide-containing fluorinated homopolymer acting as the coating layer of the nanostructure, resulting in a high level of tumor uptake and pH-activated controlled release of encapsulated DOX which were monitored via ^19^F MRI 138.

To note, non-specific pH activation could occur in normal cells since they have an acidic environment in their lysosomes or late endosomes (pH < 5.4). To achieve drug release only in the extracellular tumor microenvironment at a pH of ~6.6, but not inside normal cells, an accelerator/brake strategy was adopted to regulate the optimal pH response. A core-satellite nanomedicine, SPNs@CoOOH, was prepared: semiconducting polymer nanoparticles as the satellite layer, and cobalt hydroxide oxide nanoparticles as the core. A low pH in the extracellular tumor tissue accelerated the release of Co^3+^ ions from SPNs@CoOOH, and the released Co^3+^ ions reacted with H_2_O to generate products such as ^1^O_2_ and H^+^. H^+^ exerted a braking effect towards the chemical reaction to control the release of Co^3+^ ions, and the therapeutic ^1^O_2_ would activate the thiophene unit in the nanomedicine to generate near-infrared chemiluminescence via chemically electron exchange luminescence. By regulating the percentage of Co^3+^ and the semiconducting polymer, efficient ^1^O_2_ production and high chemiluminescence signal were realized for chemiluminescence imaging-monitoring, pH-selective cancer therapy 139.

Taken together, under different tumor acidosis conditions at extracellular, endosomal, or lysosomal locations, the protonation of the pH-sensitive moieties in the polymers and/or the dissociation of inorganic nanostructures contribute to nanostructural transformation, leading to cargo release, aggregation, disassembly, and disintegration. Precisely manipulating the response of chemical moieties to a slight change in the pH is achievable in the organic nanomedicine, thus pH-sensitive drug-conjugated organic nanomedicine is appealing from the aspects of chemistry, manufacture and control (CMC) manufacture processes and biosafety. A systematic study of inorganic nanostructural responses to a broad pH range should be pursued to understand their activatable drug release behavior and imaging/therapy effects. Meanwhile, the impact of long-term retention of these metals or micronutrients (e.g., Fe and Mn ions) in the organs after treatment with inorganic nanomedicine should be carefully investigated.

#### 3.2.2. Redox-activatable theranostic nanomedicine

In the redox-activatable nanomedicine, the breakage of a disulfide bond or other redox-sensitive ligands in response to ROS and/or GSH contributes to an enhanced theranostic performance 140. Meanwhile, the reaction of bioactive ingredients (metal nanoparticles) in the nanomedicine with redox agents enables “turn-on” imaging, chemodynamic therapy, ferroptosis, immunomodulation, and homeostasis disturbance.

**GSH-induced disassembly.** One GSH-activatable Fe-Cu@PANI nanoparticle synthesized by doping metal ions (Fe^3+^ and Cu^2+^) and aniline into a BSA template was prepared and used for PAI-guided PTT. Cu^2+^ in this nanomedicine reacted with abundant GSH in the tumor cells, leading to the formation of an emeraldine salt status of protonated PANI and a redshift to 820 nm for NIR, thus generating a cascade-like enhanced GSH response evidenced with simultaneous PA signal/localized heat. Transmission electron microscopy (TEM) images demonstrated that the size of Fe-Cu@PANI NPs significantly decreased from ~230 nm to 7 nm after incubation with 1.0 mM GSH. *In vivo* PAI further confirmed its GSH-activatable imaging property, as the PA intensity in the tumor area markedly increased after 6 days *i.t.* injection and remained stable on day 12 to day 14. Thermographic images of 4T1 tumor-bearing nude mice revealed that the temperature in the tumor area rose to 55.6 °C in the group treated with Fe-Cu@PANI + 808-nm laser irradiation, while in the group treated with PBS at the same irradiation condition, the temperature merely reached 40.3 °C, indicating a potent thermal-generation capacity of Fe-Cu@PANI. Furthermore, the relative tumor volume in the Fe-Cu@PANI + laser-treated group shrank to nearly zero on day 16 post-treatment, while it was ~6.5, ~6.5, and ~5 for the group treated with PBS, Fe-Cu@PANI, and PBS + laser, respectively 141.

This GSH-induced disassembly strategy also applies to molecular assembly-based theranostic nanomedicine. Two GSH-activatable molecular structures (1-Zn-PPA and 1-NLG) were prepared by covalently conjugating photosensitizers Zn-PPA-SH or IDO inhibitors NLG919 to the 2-Gd molecular backbone with a disulfide bond, respectively; MRI agents Gd-DOTA, hydrophobic fluorophores AO-Luc, and tumor-targeting ligands cRGD were integrated into the molecular backbone. These two molecular structures self-assembled into a spherical nanoassembly at a size of ≈ 100 nm with a high r_1_ value of 18.7 ± 0.3 mM^-1^ s^-1^ and a quenched fluorescence status. Upon exposure to GSH, the functional molecules were activated or released from the disassembled nanostructure, exerting their therapeutic or imaging effects: a) the FL intensities of AO-Luc at 547 nm and Zn-PPA-SH at 672 nm were markedly enhanced with a ~500- and ~85.9- fold improvement, respectively, b) the released NLG919 inhibited IDO1, reducing immunosuppressive Tregs, and c) the released Zn-PPA-SH could bind to albumin for robust ROS generation upon light/ultrasound stimulation, inducing a direct tumor-killing effect or augmenting immunological cell death. In treating the mice bearing orthotopic 4T1 breast cancer and GL261 glioma brain cancer, the FL/MR bimodal signal-guided synergistic sonodynamic and photodynamic therapy showed a remarkable tumor suppression rate, a robust immunogenic cell death (ICD)-mediated immune response, and an extended survival time 142.

**Figure 5:**
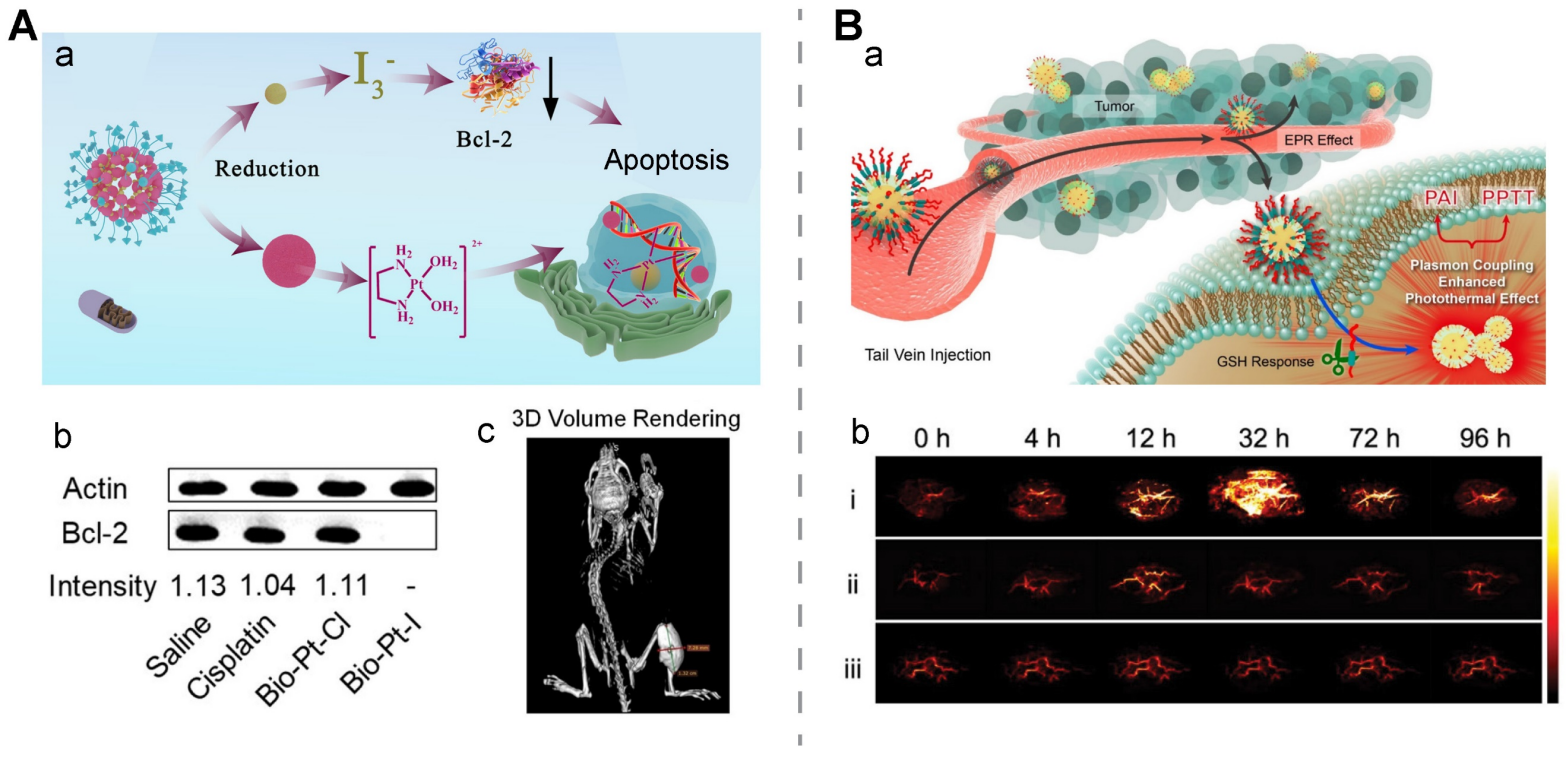
GSH-activatable nanomedicine for cancer theranostics. A) GSH-activatable iodine-conjugated Pt(IV) nanomedicine (Bio-Pt-I) for computed tomography-guided chemotherapy. a) Schematic of Bio-Pt-I design. b) Western blots of Bcl-2 expressed in tumors after various treatments. c) 3D volume-rendering CT images of PDX tumor models injected with Bio-Pt-I. Reproduced with permission 146. Copyright 2022, American Chemical Society. B) A GSH-activatable nanoprobe for photoacoustic imaging and photothermal therapy. a) Schematic design of the nanoprobe. b) PAI images of tumor sites treated with the nanoprobe (i), Au@PDA-PEGm NPs (ii), and PBS (iii) at different time points. Reproduced with permission 148. Copyright 2022, American Chemical Society.

GSH-mediated reduction of Pt(IV) prodrugs to active Pt(II) drugs is a typical example of redox-induced activation of nanomedicine for cancer theranostics 143. The use of a Pt(IV) prodrug-derived nanostructure with imaging moieties could improve the bioavailability of Pt(II) and enable imaging-assisted therapy. For example, a MnO_2_-Pt(IV) nanomedicine prepared by the one-pot ultrasonication method was reduced to Mn(II) and Pt(II) in tumor cells. These released metal ions exerted a ~4-fold cytotoxic effect against A549 cells over the Pt(IV) precursor, meanwhile, a ~2.4-fold T1-MRI enhancement was achieved at in-vivo tumor sites compared to MnCl_2_ 144. Another single molecule-assembled Pt(IV) prodrug was prepared by incorporating methylene blue, a photosensitizer, and quinone methide (QM), a GSH-depleting adjuvant, in the hydrophobic domain of the prodrug. In this design, both Pt(IV) and QM were dedicated to disrupting the cellular GSH-defense system, indirectly boosting therapeutic effects from methylene blue-mediated PDT and Pt(II)-mediated chemotherapy. *In vivo* fluorescence imaging mediated by GSH-activated methylene blue revealed peak accumulation of this prodrug nanomedicine at 8 h post-injection 145. In another study, a nanomedicine with a simple structure, Bio-I-Pt, was developed via conjugating iodine and biotin to a Pt(IV)-based small molecule in which the Pt and I content was 47.1% and 36.1%, respectively. This molecule self-assembled into spherical nanoparticles at a size of around 100 nm with a high X-ray attenuation efficiency. After internalization of the spherical nanostructure into cellular cells at a high GSH concentration via biotin-receptor interaction, Pt(IV) in the nanomedicine was reduced to Pt(II) for chemotherapy and I_3_^-^ generated from reduction helped reduce Bcl-2, thus reverting Pt-based drug resistance. After incubation with reductive sodium ascorbate for 12 h, the Pt and iodine release rates were 73.2% and 73.9%, respectively, while in the buffer without the reductant, the accumulative release ratio for Pt and iodine was 28.8% and 24.4%, respectively. TEM images suggested distinctive dissociation of the spherical nanostructure under reductive conditions. CT images of the Bio-Pt-I-treated tumor-bearing mice helped delineate the tumor area at a high density at 12 h post-intravenous injection. Western blotting results confirmed that the Bcl-2 expression intensity was 0.97 and 1.72 in the saline-treated A549 and A549/DDP cells, while the intensity sharply dropped to 0 and 0.1 in the A549 and A549/DDP cells after Bio-Pt-I intervention, respectively (**Figure 5A**) 146.

** GSH-induced assembly.** Gold nanoparticles coated with a GSH-sensitive layer are one representative nanomedicine with GSH-induced aggregation. A DNA sequence with a disulfide bridge was connected to G-quadruplex-hemin or G-quadruplex-Ce6 with AuNPs modified with a thiolated capture probe to construct Au-GH-dsDNA-DOX and Au-GC-dsDNA-DOX, respectively, after DOX was loaded onto the AuNPs. Under a reductive environment, the disulfide bridge in double-stranded DNA was broken to induce cargo release and AuNPs aggregation. GSH-triggered AuNPs aggregation after GSH intervention, which was ascribed to the hybridization of the exposed end of the capture probe, was verified through the following observations: the aggregates in the TEM images; a remarkable size increase from ~25 nm to ~500 nm in the DLS diagram; a red shift in the absorption spectra; and a dramatic temperature rise in response to light irradiation 147. It was also reported that mPEG-CONH-ss-NH_2_ was introduced to a polydopamine coating shell of AuNPs to form Au@PDA-ss-PEGm NPs, and the NPs without disulfide bonds were used as a control. Upon GSH stimulation, long-chain PEG was detached and naked Au@PDA NPs aggregated due to an imbalance in the surface charge caused by a high-concentration of salt ions. These GSH-triggered nanoaggregates helped display enhanced PA images and plasmon coupling enhanced the photothermal effect. The peak of the PA intensity in the tumor area treated with this activatable nanostructure had a 7.2-fold increase at 32 h post-injection, while there was no significant signal change in the groups treated with PBS and the NPs without the disulfide bonds. Meanwhile, a distinctive temperature rise of 38.9 °C was seen in the tumor area of the HeLa tumor-bearing mice treated with the activatable nanostructure plus laser irradiation, leading to a remarkable tumor inhibition rate, while negligible changes in the thermal imaging signal and no tumor growth inhibition were seen in the control groups (**Figure 5B**) 148.

**ROS-induced disassembly.** In response to an elevated ROS level in tumors, the most common activatable result is the disassembly of the nanomedicine, while inorganic nanostructures may perform a similar catalysis function as catalase. For example, a fluorescent probe Ag_2_S QDs and tellurium (Te) nanorods were prepared via a polypeptide PC_10_AGRD-assisted biomimetic method and then coated with extracted 4T1 tumor cell membranes to form the cancer cell membrane-coated Ag_2_S QDs and Te nanomedicine (abbreviated as CCM@AT). The NIR-II FL emission of Ag_2_S QDs was quenched by Te nanorods in the prepared nanomedicine. Meanwhile, the dissociation of TeO_6_^6-^ from the Te nanorod upon stimulation with a high H_2_O_2_ level triggered a switch-on for NIR-II imaging by Ag_2_S QDs; meanwhile, the released TeO_6_^6-^ and the exposed Ag_2_S QDs synergistically exerted a combined effect of chemotherapy and photothermal therapy. *In vivo* FL imaging of the 4T1 tumor-bearing mice displayed the tumor-specific distribution of CCM@AT, while multi-organ distribution (liver and spleen) of the nanostructures without the Te nanorod indirectly indicated the vital role of the redox-activatable Te nanorod. As a consequence, the treatment with CCM@AT and laser outperformed other treatments, with a high tumor inhibition rate of 98.4% in comparison with 65.3% for CCM@AT without laser, 68.9% for CCM@T, and 86.9% for CCM@A and laser 149.

**Figure 6:**
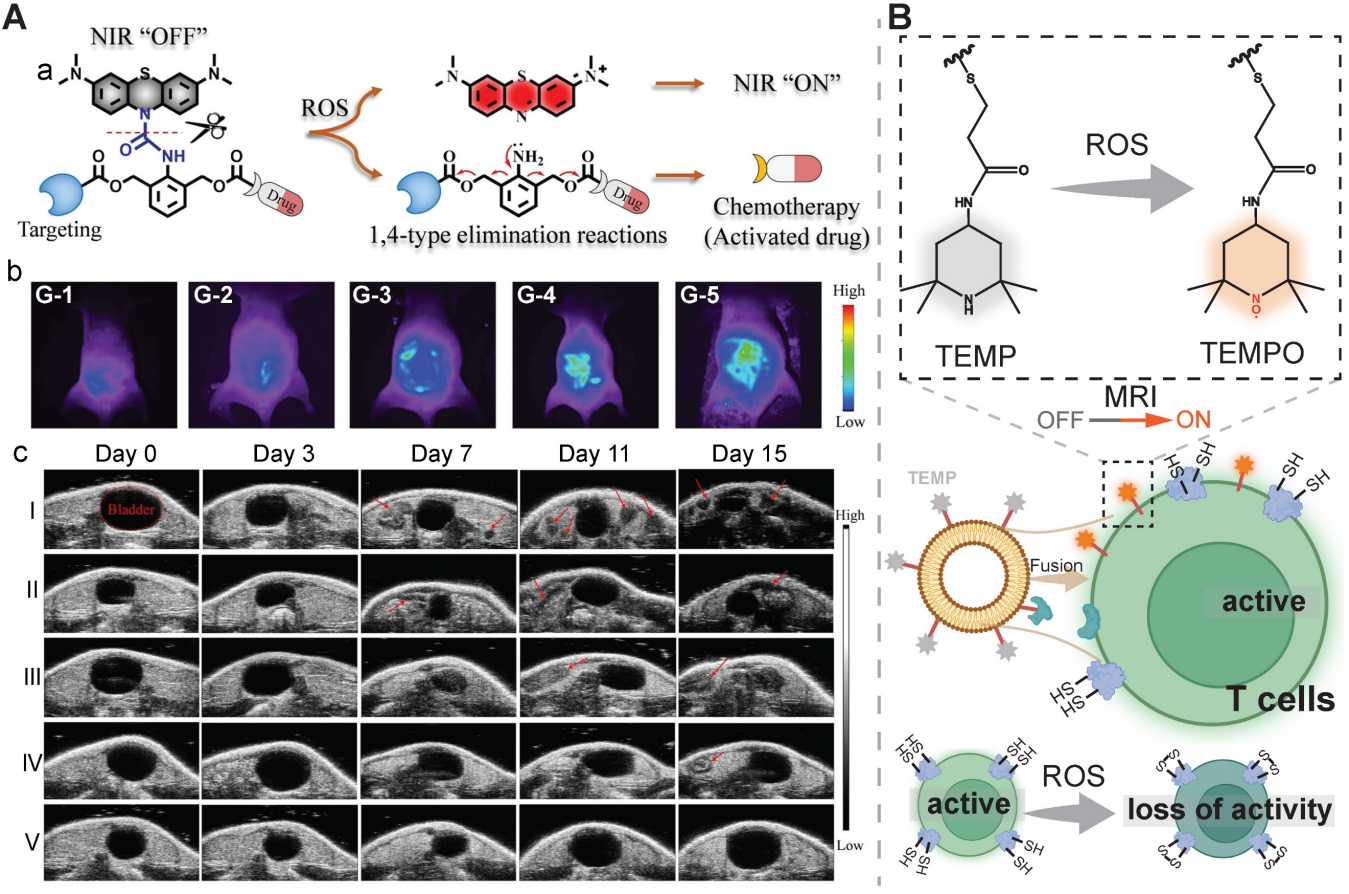
ROS-activatable nanomedicine for cancer theranostics. A) An ROS-activatable prodrug nanomedicine for chemotherapy and NIR imaging of cervical cancer metastases. a) Illustration of ROS-activated disassembly of the prodrug nanomedicine. b) Fluorescence images of cervical cancer metastases at different stages with the aid of this nanomedicine. c) Long-term ultrasound imaging of the mice treated with different formulations (I: saline; II: free CPT; III: (4)-MB-CPT, IV: (8)-MB-CPT; V: Bio-(8)-MB-CPT). CPT: camptothecin; MB: methylene blue; Bio: biotin. Reproduced with permission 151. Copyright 2022, Wiley-VCH. B) ROS-induced on-site activation for immunotheranostics. 2,2,6,6-tetramethylpiperidine (TEMP)-modified liposome was fused with T cells via active targeting. When these fused T cells were exposed to an elevated ROS level in the TME, the TEMP on the liposome surface was oxidized to TEMPO. This procedure scavenged intracellular ROS to restore the antitumor activity of T cells and enable a turn-on of MR imaging for monitoring the therapeutic response. The figure was created with BioRender.com according to the hypothesis presented in Ref 152.

Redox-triggered disassembly of organic nanomedicine could result in a “turn-on” of optical imaging due to the disruption of aggregation-caused quenching or Förster resonance energy transfer. A fluorescently traceable prodrug nanomedicine was prepared by bridging zwitterionic fluorescent rhodamine (RhB) with chemotherapeutic camptothecin (CPT) via a disulfide bond. Interestingly, the responsiveness of the nanomedicine towards both H_2_O_2_ and GSH was verified with a rapid fluorescence enhancement was observed at the condition of ≥ 100 mM H_2_O_2_ and ≥ 10 mM GSH. With the aid of this redox-cleavable linkage, a high tumor inhibition rate of 77.4% was achieved in comparison with 51.2% in the free CPT-treated group, meanwhile, the highest fluorescence intensity was found at tumor sites at 12 h post-injection 150. In another report, a ROS-activatable chemotheranostic prodrug nanomedicine (Bio-(8)-MB-CPT) was developed by conjugating a ROS-responsive leucomethylene (LMB) derivative to a self-immolative space-containing biotin-camptothecin conjugate. Upon exposure to ROS, LMB was converted to methylene blue fluorophores to turn on NIR imaging and CPT was released from the nanomedicine. Fluorescent imaging mediated by activated methylene blue showed a similar detection ability as ultrasonography for tracking cervical cancer abdominal metastases at different stages in murine tumor models. As shown in high-performance liquid chromatography (HPLC) results, this nanosystem exhibited a drug release efficiency of up to 92.04% after incubation with 5.0 equivalents of HOCl. Accordingly, this Bio-(8)-MB-CPT nanomedicine displayed a high tumor inhibition rate of 99.9% in treating metastatic cervical cancer and no abdominal metastases sites were found through monitoring via ultrasonography (**Figure 6A**) 151.

**ROS-induced on-site activation.** Fusing ROS-activatable (scavenging) imaging agents into therapeutic T cells is a nanoengineering strategy to realize imaging-guided immunotherapy (**Figure 6B**). A fusogenic liposome modified with αCD3 and 2,2,6,6-tetramethylpiperidine (TEMP) moieties was employed to target and fuse with T cells to produce the nanomedicine T-Fulips, and Iso-Fulips without the targeting function was set as a control. Upon stimulation by the tumoral ROS, the TEMP groups were oxidated into paramagnetic 2,2,6,6-tetramethylpiperidine 1-oxyl (TEMPO) radicals, neutralizing ROS for restoring the T cell function and allowing MRI for real-time monitoring. *In vitro* T_1_ images supported that the imaging contrast of the T-Fulips solution was substantially elevated after co-incubation with H_2_O_2_ at an increased concentration; the imaging contrast in the T-Fulips solution with 50 μM H_2_O_2_ was improved two times higher than that of the control group with Iso-Fulips. Flow cytometry analysis suggested upon the treatment with H_2_O_2_, the surface -SH groups on the T cells in the presence of T-Fulips was reduced from 21.5% to 14.1%, while it remained to be 29.7% in the group of H_2_O_2_ + Iso-Fulips. Moreover, the proliferation capacity of T cells was restored in the presence of T-Fulips. Under oxidative stress induced by X-ray, the presence of T-Fulips increased the number of CD4^+^ cells, CD8^+^ cells, and -SH+ cells in the tumor slices. These experiment results suggested T-Fulips could enhance T cells activity by regulating the -SH surface groups. The radiation therapy-treated 4T1 tumor-bearing mice were used to examine the role of T-Fulips in alleviating oxidative stress based on the visible T cells activity. Compared to Iso-Fulips without the T cells-targeting function, the presence of T-Fulips contributed to a much significant increase in the T1 signal change in the tumor area, a rise in the percentage of effector T cells (CD45^+^CD4^+^IFN-γ^+^, CD45^+^CD8^+^IFN-γ^+^, CD45^+^CD4^+^IFN-γ^+^SH^+^, and CD45^+^CD8^+^IFN-γ^+^SH^+^), as well as a superior tumor growth suppression effect. Additionally, an enhanced therapeutic outcome of adoptive T-cell therapy was seen with the aid of T-Fulips in the B16F10-OVA tumor-bearing mice. To conclude, this nano-fusion strategy in response to ROS was able to achieve both alleviating the oxidative stress and realizing real-time monitoring of T cells activities 152.

In addition to triggering drug release or activating imaging at a high tumoral redox level, modifications of the organic or inorganic nanostructures were explored to consume or covert excess redox agents, contributing to chemodynamic therapy, glutathione depletion-enhanced ferroptosis, and oxidative stress relief. Immune checkpoint inhibitors (ICIs)-based immunotherapy recently enjoys a flourishing boom, however, the potential detrimental effect of tumoral reductive agents on ICIs remains to be revealed. It is hypothesized that the disulfide bond of antibodies may be broken after exposure to GSH, resulting in a collapse in the ICI hierarchical structure. In this context, the nanoengineering approach could be explored to address the redox stress to aid in immunotherapy. Furthermore, it is encouraged to develop redox-activatable imaging for real-time monitoring of immunotherapeutic agents or cells.

#### 3.2.3. Enzyme-activatable theranostic nanomedicine

Over-expressed or tumor-specific enzymes are located at different sites of the tumor microenvironment. Utilization of these enzymes to prepare enzyme-activatable theranostic nanomedicine offers site-specific release or activation of therapeutic/imaging agents.

**Figure 7:**
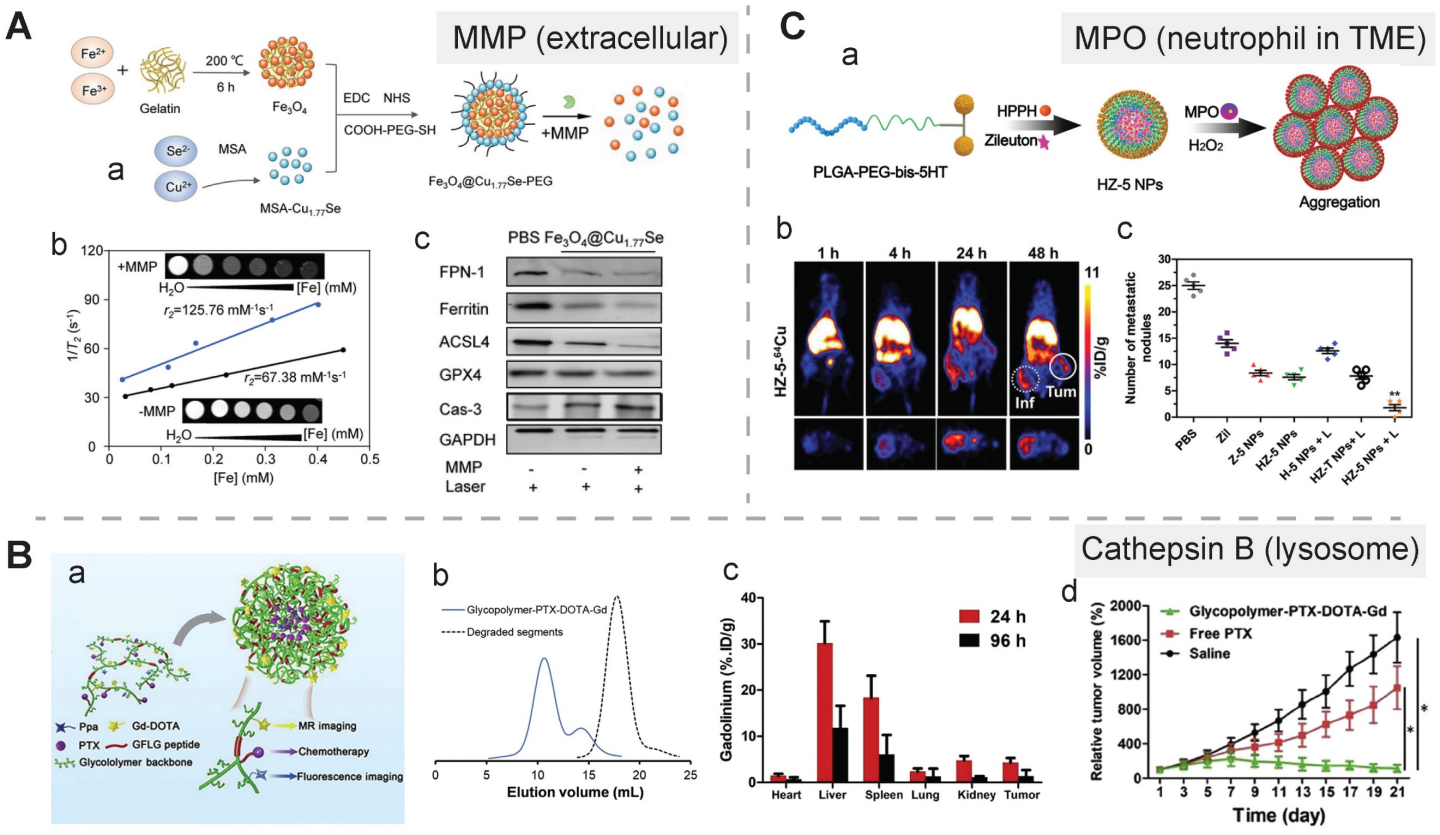
Enzyme-activatable nanomedicine for cancer theranostics. A) MMP2-induced disassembly of a nanozyme for PAI, MRI, and photothermal ferroptosis therapy. a) Schematic of the fabrication of the nanozyme and its disassembly in response to MMP2. b) T2-weighted MR images and corresponding plots of the nanozyme incubated with or without MMP2. c) Western blots analysis of ferroptosis- and apoptosis-related protein expression in 4T1 cells after different treatments. Reproduced with permission 153. Copyright 2022, Wiley-VCH. B) Cathepsin B-induced degradation of gadolinium-labeled branched glycopolymer-PTX conjugates for MRI, fluorescence imaging, and chemotherapy. a) Schematic design. b) Size-exclusion chromatography profiles for this conjugate before and after incubation with cathepsin B. c) Distribution of this conjugate in major organs determined by the gadolinium retention. d) Tumor growth curves after different treatments. Reproduced with permission 154. Copyright 2021 the Authors, published by Elsevier B.V. C) Myeloperoxidase-induced aggregation of HZ-5 NPs for neutrophil-targeting PDT or PET. a) Schematic of the aggregation process of HZ-5 NPs. b) PET images of 4T1 tumors after *i.v.* injection of ^64^Cu-labeled HZ-5 NPs. e) The counts of lung metastatic noduli in the mice treated with different methods. Reproduced with permission 155. Copyright 2020, Wiley-VCH.

**Extracellular location.** The matrix metalloproteinase (MMP) family constitutes the majority of extracellular enzymes in the tumor site. Gelatin was selected as an MMP-2 activatable linker in a theranostic nanozyme which could be disassembled in response to the extracellular enzyme. In this study, gelatin as a mediator was mixed with Fe^3+^ ions to form interlaced Fe_3_O_4_ aggregates under 200 °C, and the aggregates were subsequently conjugated with Cu_1.77_Se and modified with a PEG coating layer, resulting in the Fe_3_O_4_@Cu_1.77_Se-PEG nanomedicine with a photothermal conversion efficacy of 67.6%. MMP-2 induced distinctive disassembly of this nanostructure, and its size significantly shrank from 124.2 nm to 14.6 ± 6.9 nm, which was accompanied with an elevated level of T2 MRI contrast, an increase in the r_2_ value from 67.38 to 125.76 mM^-1^s^-1^, and an enhanced photothermal therapeutic effect due to deep penetration and an improved ferrotherapeutic efficacy. Additionally, the synergistic antitumor effect, which was due to PTT-induced caspase-3-mediated apoptosis, escalated Fenton reaction-mediated ferroptosis induced by released Fe^3+^/Cu^2+^, and immunomodulation induced by apoptosis and ferroptosis (polarizing TAMs and inducing ICD), led to effective control of both primary and distant tumors. After photoirradiation of primary tumors, the relative tumor volume in the Fe_3_O_4_@Cu_1.77_Se-PEG-treated mice was reduced to nearly zero, while it was ~8, ~5, and ~2.5 in the group treated with PBS, Fe_3_O_4_, and Cu_1.77_Se, respectively. Photoirradiation was also applied to distant tumors, and the tumor-bearing mice group treated with Fe_3_O_4_@Cu_1.77_Se-PEG displayed a remarkable decline in the relative tumor volume, and the volume was 3.9-, 3.0-, and 1.8-fold lower than that in the group treated with PBS, Fe_3_O_4_, and Cu_1.77_Se, respectively. Besides, a significant drop in the MRI intensity in the tumor site indicated this nanoprobe could spatially-temporally monitor the therapeutic process (**Figure 7A**) 153.

In addition to activation of nanomedicine by MMP2, MMP9 is often harnessed to prepare activatable organic coatings for inorganic nanomedicine. For instance, an MMP9-activatable peptide-containing polymer, PIX-(GPLGL-PEG)_2_ (abbreviated as PMP), was employed to encapsulate DOX and ultrasmall superparamagnetic iron oxide (USPIO), resulting in the PMP@USPIO/DOX nanomedicine through a self-assembly process. Upon exposure to MMP9, this nanostructure was disassembled and the imaging power was switched on. Thus, this nanostructure could be used to quantify the MMP level and monitor the drug release process 156. Another surface modification strategy by applying an MMP-9 activatable zwitterionic tetrapeptide, EG_8_-GPKGLRGD-EG_5_-C, was recently proposed to promote self-assembly of gold nanoparticles. The remaining RGD sequence after enzymatic cleavage facilitated self-assembly of gold nanoparticles with a size of 585 ± 19 nm via electrostatic interaction, which was confirmed from the TEM images of MMP9-containing NPs and MDA-MB-231 tumor cells after incubation with the gold nanoparticles 157.

**Cellular surface and intracellular location.** A group of tumor-associated enzymes are over-expressed on the cellular membrane surface or in the intracellular environment. Our group has devoted to the development of cathepsin B-responsive biodegradable theranostic nanostructures via a tetrapeptide GFLG linker. We have prepared a biodegradable branched polymeric nanoparticle, pHPMA-PTX-Gd-Cy5.5, in which cathepsin B-activatable linkers were installed on the pHPMA backbone and at the conjugated site of PTX. Size-exclusion chromatography (SEC) profiles of this nanostructure incubated with cathepsin B for various time points revealed that the molecular weight of this nanostructure remarkably dropped from 186 kDa to 25 kDa, and the PDI from 2.30 to 1.2, at 12 h post-incubation, while in the PBS-incubated group, the MW and PDI values remain unchanged, indicating cathepsin B could successfully induce the degradation of the nanostructure into small fragments for renal excretion. Conjugation with a fluorescent molecule, Cyanine 5.5, and an MR agent, gadolinium chelates, offered dual-modal imaging which helped reveal the biodistribution of the nanomedicine, monitor the processes of cellular internalization, vascular extravasation, and tumor penetration, as well as assess its tumor accumulation and evaluate its therapeutic efficacy. Moreover, the conjugated PTX could be released specifically triggered by cathepsin B, which was confirmed from the HPLC, and the released PTX decreased the tumor cell viability, damaged the microfilaments (α/β tubulin and pan‐actin), and induced apoptosis. In this study, the T1 value in the tumor area determined by T1 mapping was used for three-week monitoring of the therapeutic outcomes after various treatments 158. Next, we prepared another cathepsin B-activatable gadolinium-labeled branched glycopolymer-PTX by following the prior design concept of biodegradability. The SEC profiles indicated that after co-incubation with cathepsin B, the nanostructure (MW = 244 kDa, PDI = 2.48) was shattered to renal-excretable fragments (MW = 28 kDa, PDI = 1.26). Meanwhile, this nanostructure had an improved antitumor efficacy (tumor growth inhibition (TGI): 90.6%) and an enhanced MRI performance (r_1_ = 7.1 mM^-1^s^-1^, the peak of the relative enhanced signal intensity (SI) in the tumor: 275%) in contrast to the previous one (TGI: 81.3%, r_1_ = 8.6 mM^-1^s^-1^, the peak of SI in the tumor: ~230%) (**Figure 7B**) 154. These studies validate the feasibility of using GFLG peptides as a cathepsin B-activatable linker and provide insights into the design of enzyme-activatable polymer-drug conjugates for theranostic application 159.

Moreover, sequential enzyme activation strategy, a dual-lock activation approach for more specific drug release or a sequential turn-on of the imaging function, has been proposed for developing nanomedicine for cancer theranostics. This strategy was demonstrated by a recent study with a theranostic probe, PLCy, which was prepared by conjugating PEG to a mitochondria-targeting NIR fluorophore, hemicyanine (CyNH_2_), via an acetylated lysine Boc-Lys(Ac)-OH group sensitive to two enzymes. The theranostic function of CyNH_2_ was masked via a twisting intramolecular charge transfer effect. Sequential stimulation by histone deacetylase and cathepsin L assisted in the detachment of PEG and exposure of CyNH_2_, leading to a switch-on of fluorescent imaging of tumor cells and exertion of the NIR phototoxicity on mitochondria, respectively 160.

**TME-infiltrated neutrophils.** Neutrophils-associated enzyme activation strategy applies to the design of cancer nanomedicine for theranostics, which may be helpful in the case of cancer-associated inflammation. The example for this strategy was demonstrated with myeloperoxidase (MPO) in neutrophils as a targeting biomarker and an endogenous stimulus for triggering nanoparticle aggregation. The ligand modified with two 5-hydroxytryptamine ends, abbreviated as bis-5HT, reacted with MPO to produce radicals that could bind to other residues for prolonged retention of bis-5HT in the neutrophils. To apply bis-5HT to theranostics of neutrophils, it was conjugated to PLGA-PEG-COOH in which a leukotriene inhibitor, zileuton, and a photosensitizer, HPPH, were encapsulated, resulting in activatable HZ-5 NPs. Upon co-incubation of HZ-5 NPs with a MPO buffer or 4T1 cells, nanoaggregates could be seen in the TEM images and confocal images, and an increase in the size from 105 ± 15 to 246 ± 33 nm in the DLS diagram. To perform imaging-guided therapy, ^64^Cu-labelled HZ-5 NPs reached the highest level of tumor accumulation at 24 h post-injection, and this time point was determined to be an optimal time for performing PDT. Moreover, the uptake of these theranostic nanoagents increased from 4.8 to 7.7 ID g^-1^ after the PDT intervention, indicating its auxiliary function was to monitor PDT-induced cancer inflammation. Moreover, neutrophil-induced lung metastasis was effectively inhibited in the tumor-bearing group treated with HZ-5 NPs + laser, confirmed by FL images in the mice and photographs of the excised lung tissues (**Figure 7C**) 155.

Overall, the theranostic effects of the enzyme-activatable nanomedicine rely on the location of enzymes, either forming a self-assembly nanoaggregate or inducing disassembly of its nanostructure. In addition, it is worth noting that the biological function of enzymes that are actively involved in the therapy (e.g., PDT, PTT, and RT) should be carefully identified and their corresponding activatable ligands could be constructed for specific theranostic performance.

#### 3.2.4. Other endogenous stimuli-activatable theranostic nanomedicine

Hypoxia confers drug resistance to various cancer therapies, and it emerges as a potential theranostic target. Ideally, a hypoxia-activatable theranostic agent should aid in monitoring the therapeutic response of hypoxia-activatable prodrugs by real-time delineating the hypoxia area and indicating the hypoxia degree. In one study, a chemotherapeutic CPT drug was connected to boron dipyrromethene (BODIPY), a fluorescent photothermal sensitizer, via a hypoxia-responsive azobenzene linker, forming a heterotrimer prodrug BAC. A hypoxia-activatable theranostic nanostructure, HSA@BAC, was prepared after loading BAC into human serum albumin (HSA). In this nanostructure, fluorescent emission of BODIPY was quenched by CPT via fluorescence resonance energy transfer (FRET). Upon exposure to overexpressed azoreductase in the tumor hypoxia area, this nanostructure was disassembled to release the CPT drug and turn on fluorescence imaging, which provided guidance for precise, localized light irradiation 161. A dual-emissive Pt^II^ metallacage with hypoxia-activated red phosphorescence and steady blue fluorescence was coated with an amphiphilic diblock copolymer (mPEG-*b*-PBLG) to act as a hypoxia-activatable theranostic nanoagent. Heteroligation directed self-assembly of Pt^II^-*meso*-tetra(4-carboxyphenyl)porphine as an oxygen-sensitive phosphorescent ligand, 9,10-di(pyridin-4-yl)anthracene that emits blue fluorescence, and a Pt^II^ acceptor to form an activatable metallacage. After incubation of the mPEG-*b*-PBLG-coated metallacage with 4T1 cells under normoxia and hypoxia conditions, a significant enhancement of up to around 450% in red phosphorescence and an increased red/blue emission ratio from 0.224 to 0.886 were observed in the confocal laser scanning microscopy (CLSM) images of the hypoxia-treated cells. Meanwhile, three agents including the Pt^II^ metallacage, the mPEG-*b*-PBLG-coated Pt^II^ metallacage, and free cisplatin were demonstrated to exert similar cytotoxicity towards 4T1 and A549 cells. However, high tumor accumulation of mPEG-*b*-PBLG-coated Pt^II^ metallacage contributed to a better and safer antitumor performance compared to free cisplatin, which was indicated by blood biochemistry tests, body weight measurements, and tumor volume changes 162.

Biological gasotransmitters, such as CO, NO, and H_2_S, may be overexpressed in the disease sites. These gasotransmitters may aid in the transformation of the nanomedicine for an enhanced therapy/imaging effect. For instance, intratumoral hydrogen sulfide (H_2_S) has been reported to convert AgNPs into Ag_2_S NPs, which exerted a photothermal effect for therapy and offered near-infrared imaging 50, or react with Fe^3+^ ions released from a nanostructure to produce Fe_1-x_S for MRI-guided PTT 163. One H_2_S-activatable theranostic nanoagent exhibited NIR conversion from 1070 nm to 720 nm and ratiometric PA signal responsiveness with stable PA_680_ signal and weakened PA_900_ signal upon H_2_S stimulation. During this activation process, fluorophore ZM1068-NB was converted into ZM1068-Ketone with the consumption of H_2_S. Furthermore, the depletion of H_2_S combined with the photodynamic effect of activated ZM1068 contributed to the improved antitumor performance in a colorectal tumor-bearing mice model via apoptosis 164.

Taken together, the over-expressed or specifically-produced agents in tumor cells can be leveraged as endogenous stimuli for developing activatable nanomedicine for cancer theranostics. The responsiveness of the activatable linkers towards these stimuli could be dramatically different due to the preparation procedure of the nanomedicine, the stimuli type, and the constitutional components of the tumor microenvironment. A couple of activatable linkers for the same stimulus should be evaluated for the nanomedicine, particularly drug-polymer nanoconjugates, and the variations in the responsiveness should be unveiled after the application of the nanomedicine to human- or murine-derived cancer cell lines and their corresponding animal models.

#### 3.2.5. Exogeneous stimuli-activatable theranostic nanomedicine

Exogenous stimuli (light, ultrasound, magnetic field, X-ray) as physical forces or activation sources have the advantages of spatial and remote control for activating cancer theranostics 165, 166. More importantly, radiosensitizers/photosensitizers/sonosensitizers/photothermal agents with a high conversion efficiency are preferred because the drug dosage and the exogenous stimuli dosage could be significantly reduced. To note, exogeneous physical stimulation via mechanical forces (compression, tension, shear force, and torque) could aid in cancer therapy by enhancing vascular permeability, imposing mechanical stress on the TME, or transiently relieving the blood-brain barrier 167.

**Light.** Light directly triggers drug release, activates PDT/PTT agents, or enables PAI. In this context, FLI-guided PDT and PAI-guided PTT have been realized. The optimal duration for light irradiation is usually determined by the time to reach the highest tumor accumulation of the nanomedicine, indicated by imaging signal or contrast.

**Figure 8:**
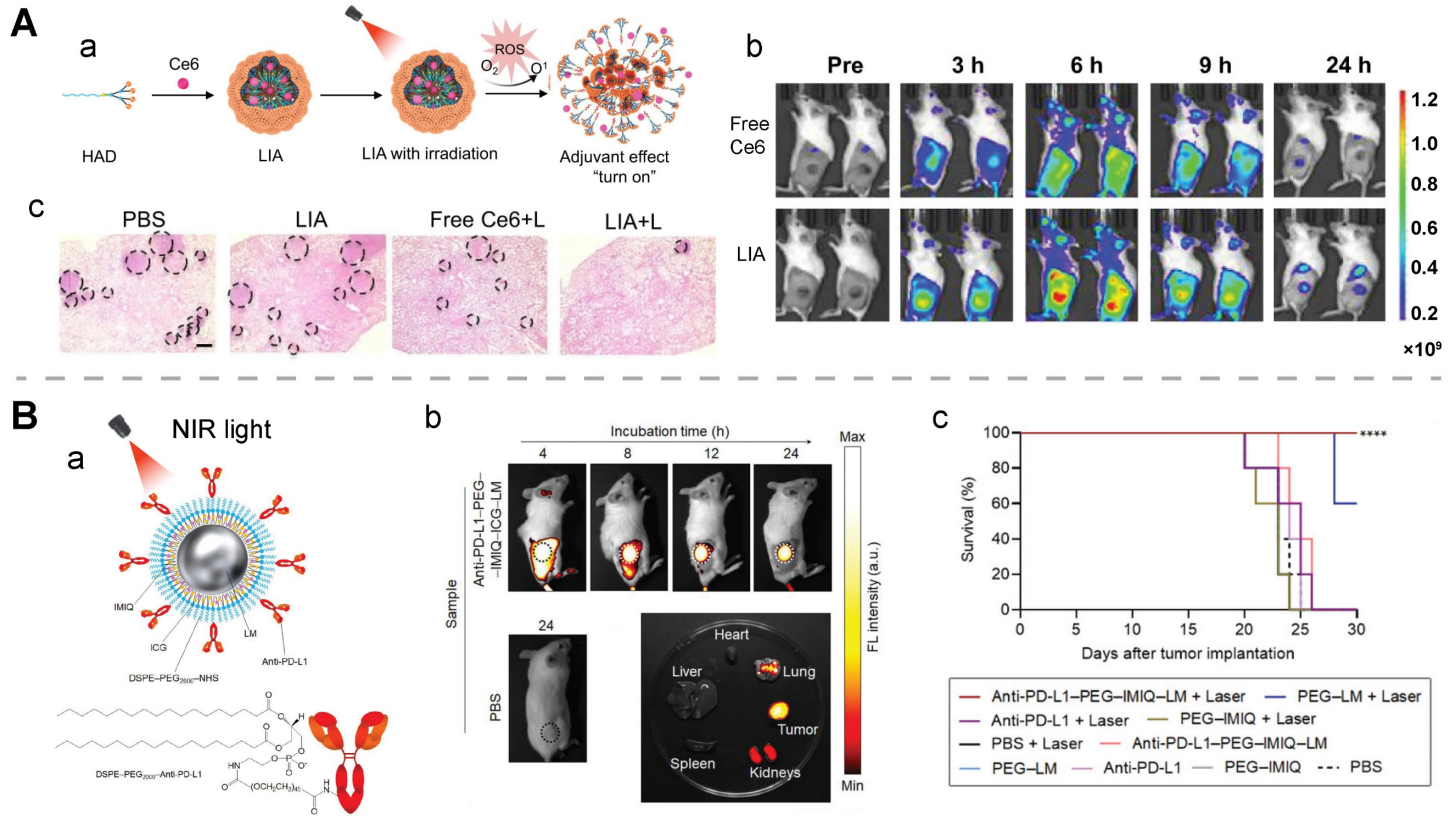
Light-activatable nanomedicine for cancer theranostics. A) A light-activatable amphiphilic dendrimer, LIA, as a fluorescence imaging-assisted immunological adjuvant. a) Schematic of the procedure for preparing LIA and triggering its adjuvant function. b) Fluorescence images of 4T1 tumor models treated with free Ce6 and LIA. c) H&E staining of lung tissues after different treatments, and black circles represented metastatic nodules, scale bar: 500 μm. Reproduced with permission 168. Copyright 2021 the Authors, published by Springer Nature. B) A light-responsive liquid metal (LM)-based immunostimulator for NIR-light triggered PTT and release of immunomodulators (anti-PD-L1 and imiquimod (IMIQ)). a) Scheme for preparation and structure of anti-PD-L1-PEG-IMIQ-ICG-LM. b) Fluorescence images of CT26-bearing mice and their resected organs after injection of this theranostic nanomedicine. c) Survival rates of the murine tumor model after different treatments. Reproduced with permission 169. Copyright 2023, published by Wiley-VCH.

It has been reported that oxygen is heavily consumed during a photodynamic therapeutic procedure, leading to a hypoxia environment. This finding was utilized to prepare a light-activatable immune adjuvant, denoted as LIA. In this design, PDT-induced hypoxia could aid in the structural transformation of the nanomedicine for eliciting *in situ* vaccination. The loaded chlorin e6 (Ce6) simultaneously acted as a photodynamic agent for therapeutic function and a fluorescent indicator for assessing tumor accumulation. Ce6-mediated PDT was utilized to damage tumors to release antigens, meanwhile, tumor hypoxia induced by oxygen consumption during PDT aided in transforming the 2-nitroimidazole group in dendrimers to 2-aminoimidazole, exerting an rHAD-mediated adjuvant effect. The reduced oxygen concentration and an increased phosphorescence lifetime or a boosted Singlet Oxygen Sensor Green (SOSG) intensity confirmed the hypoxia environment induced from the combined treatment with LIA and laser irradiation. TEM images revealed time-dependent structural collapse of LIA upon exposure to light, supporting its light-activatable disassembly behavior. Furthermore, in a 4T1 residues-bone marrow-derived dendritic cells (BMDCs) co-incubation model, a nearly two-fold increase in the mature CD80^+^CD86^+^ BMDCs was seen in the group after the treatment with LIA and light irradiation. While there was no change in the number of mature CD80^+^CD86^+^ BMDCs in other treatment groups, including PBS, free Ce6, and Ce6-containing DSPE-PEG_2k_, with or without light irradiation. Transcriptomic analysis and molecular docking experiments suggested the rHAD-induced DC maturation was realized via the toll-like receptor 7 (TLR7) pathway. Consequently, this light-activated theranostic nanoagent exhibited a superior therapeutic efficacy on primary tumors, abscopal tumors, and rechallenged tumors (**Figure 8A**) 168.

In addition to activating PAI and inducing PTT, NIR light functions as a drug-release trigger on photothermal nanoagents. In one study, Ag_2_S QDs and chemotherapeutic DOX were camouflaged by macrophage-derived extracellular vesicles via electroporation. NIRF imaging of the tumor-bearing mice treated with this camouflaged nanostructure revealed a duration of 24 h to reach a peak in its tumor accumulation, and the amount of the nanostructure in the tumor was determined to be 16.5% ID/g by inductively coupled plasma mass spectrometry (ICP-MS) analysis. Notably, light in this case was a potent trigger for drug release and structure decomposition. Upon 808 nm light irradiation, the accumulative release of both drugs was significantly improved: the DOX release rate increased from 9.6% to 58.5%, and the QDs release rate rose from 2.1% to 47.0%. Treatment with the light-activated nanomedicine resulted in a high tumor inhibition rate of 86% and an Ag_2_S QDs clearance efficiency of > 90% 170. In another study, NIR-II light acted as a trigger to release carbon monoxide (CO) from Pd@PdCO-MOF, a photo-activated theranostic agent which was composed of a porphyrin-palladium MOF shell and a palladium nanosheet core. Its PCE was found to be 44.6 % *in vitro*. The CO release result revealed that the release amount reached a peak value of 14 μM after 1 h light irradiation, and the fluorescent intensity of COP-1 (a CO fluorescent probe) in the CLSM images of 4T1 cells substantially increased with an increase in the irradiation time in the first 10 minutes. The released CO gas was found to enhance PTT through decreasing the ATP level, downregulating HSP expression, and upregulating the expression of cyto C 171. Similarly, NIR light was reported to induce the release of immunomodulators (anti-PD-L1 and imiquimod) and fluorescent molecules from eutectic gallium-indium liquid metal nanoparticles, a potent PTT agent with a PCE of 47%. During the FL-guided photothermal immunotheranostics of a murine colon cancer model, the treatment with the light-activatable nanomedicine + laser exhibited a superior tumor control rate (nearly complete control) and a significantly higher survival rate than other treatments including PBS, PEG-LM, anti-PD-L1, PEG-IMIQ, and their combination with laser. Statistically, the survival rate of the CT26-bearing mice group treated by this light-activatable nanomedicine + laser reached 100%, while the survival rate dropped to 60% in the group treated with PEG-LM + laser and 0% for the other groups (**Figure 8B**) 169. Another study further confirmed light-activatable drug release from a photothermal immunothreanostic nanomedicine, AuPB@PDA/Mn. The immunomodulator Mn^2+^ ions were loaded in the polydopamine coating of plasmonic gold blackbody. And upon NIR-II-light, Mn^2+^ ions were discharged by local hyperthermia generated by AuPB@PDA 105.

Overall, light is commonly used as an exogenous stimulus in the cancer theranostic application. Nanostructures with a NIR-II light (1000-1700 nm)-activated photothermal and photoacoustic performance could be explored to address the issue of poor tumor penetration by routine light therapy. Moreover, the photothermal or photodynamic conversion efficiency of a nanostructure is essential for the therapeutic outcome of the nanostructure-derived nanomedicine. Various design strategies on the nanostructure, such as engineering of dual-acceptor semiconductor polymers and modulating donor/acceptor groups, have been exploited to improve the conversion efficiency and imaging performance 172, 173. In addition, Janus-like PTT NPs were reported to significantly increase local temperature than conventional ones due to their thermophoresis effects 174, and the generated heat gradients could drive Janus-like NPs to deep tumor sites via a thermophoretic force 175.

**Ultrasound.** Ultrasound-activated theranostics are a combination of sonodynamic therapy 176, high-intensity focused ultrasound-induced thermal damage, and ultrasound-augmented imaging 177. Additionally, ultrasound, particularly focused ultrasound, can be exploited to trigger drug release from the theranostic nanomedicine through an ultrasound-induced sonoporation effect or ultrasound-assisted microbubble destruction. An activatable Au-DNA nanoswitch was explored to realize ultrasound-triggered drug release. Double-stranded DNA as a mechanophore was bridged between two gold nanoparticles, and the resulting nanostructure was then loaded with DOX to obtain an ultrasound-activatable nanomedicine. As observed in the TEM images, the percentage of closed dimers significantly decreased, while single particles or aggregated particles steadily increased with an increase in the ultrasonication time. Besides, the open-dimer configuration appeared at 10 min post-ultrasonication and it was then reduced. These TEM images combined with rapid drug release from the ultrasonication-treated Au-DNA nanoswitch unveiled drug release was induced from ultrasound-triggered force-stretched activation of the nanomedicine 178. In another study, high-intensity focused ultrasound (HIFU) was employed to cleave 4,40-azobis (4-cyanovaleric acid) (ACVA) C-N bonds between the MnFe_2_O_4_@CoFe_2_O_4_ core and the 1-adamantylamine-β-cyclodextrin cap to release the caged chemotherapeutic DOX. This de-capping release process was real-time monitored via enhanced MR contrast induced by an increased level of water around the magnetic nanoparticle core 87. MR-guided focused ultrasound (MRgFUS) is currently used to treat mental disorders and it has shown promise in the application of cancer theranostics. The thermal effect induced by focused ultrasound can be employed to trigger controlled release of therapeutic drugs from the nanomedicine and offer thermal images of the tumor tissue. L-menthol as a thermal-sensitive valve, DOX, and Fe ions were incorporated into mesoporous organosilica nanoparticles as an ultrasound-induced thermal-activatable theranostic nanoagent. In this nanoagent, Fe-mediated T2WI revealed enhanced tumor accumulation of the nanotheranostic agent. MRgFUS induced a hyperthermia condition (45 °C) to open the L-menthol valve for robust DOX release 179.

To conclude, with the aid of sonosensitizers, low-energy ultrasound can be a preferable and safe choice for removing a cap or loosening a nanostructure to release incorporated drugs. In addition, the design of sonosensitizers is critical. For example, one recent study demonstrated that a protein coating on hydrophobic MSN nanoparticles could significantly increase the cavitation activity in contrast to coatings with F127 polymers or phospholipids 180.

**Figure 9:**
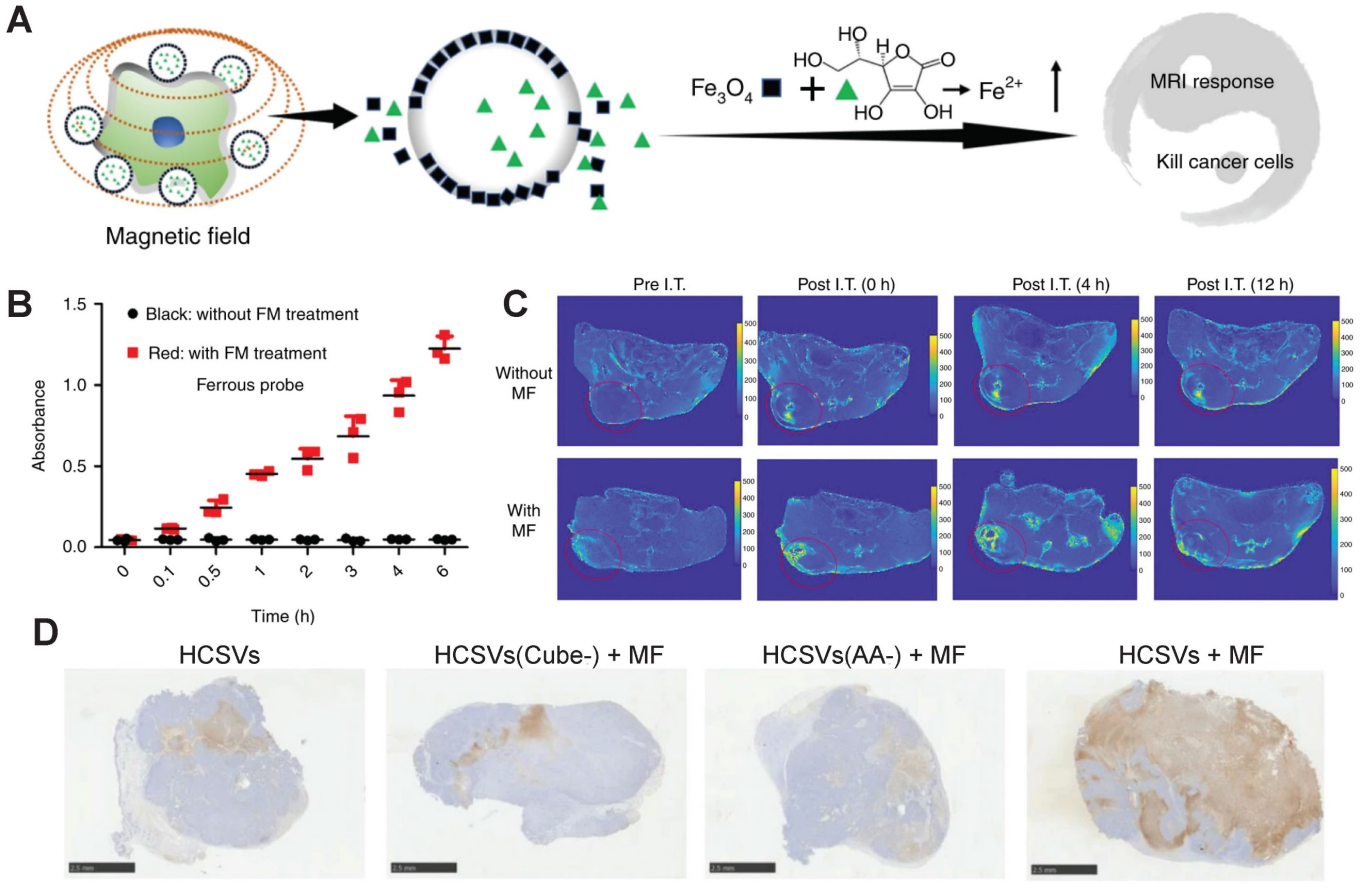
Magnetic field-activatable nanomedicine for cancer theranostics. Magnetic field-activatable hybrid core-shell vesicles for ferroptosis-like cell death-mediated immunotherapy and MRI. A) Schematic of the vesicles for MRI and therapy in a magnetic field. B) Change in the concentration of ferrous ions that were released from this vesicle with/without a FM at different time points. C) *In vivo* T2 mapping of the tumor site after* i.t.* injection of the nanovesicles with or without a magnetic field. D) TUNEL-stained tumor slices of the TRAMP-C1 model after different treatments, scale bar: 2.5 mm. Reproduced with permission 181. Copyright 2020 the Authors, published by Springer Nature.

**Magnetic field.** An exogenous circularly polarized magnetic field (MF) may act as an exogenous stimulus for ferrous metal-based nanoagents. A hybrid core-shell vesicle (HCSV), consisting of an ascorbic acid-containing core and an iron oxide nanocube-embedded PLGA shell, was reported to induce ferroptosis-like immune response-mediated cell death and activatable MRI under a MF. The MF destroyed the shell via a forced circular back-and-forth movement, and the released ascorbic acid in the core reacted with Fe_3_O_4_ to convert ferric to ferrous via the Fenton reaction. This was confirmed with a cube-like empty structure in the TEM images, a substantial increase in the absorbance of ferrous ions, a steady decline in the TRAMP-C1 tumor cell viability, and a significant increase in the CRT exposure (57.5 ± 2.7 %) after the TRAMP-C1 tumor cells were treated with HCSVs in a MF. An activated MRI performance was supported with a significant reduction in the r_2_ value of IONCs from 13.90 to 0.57 mM^-1^s^-1^ when incubated with ascorbic acid, and a remarkable boost in the T2 signal in the tumor area after *i.t.* injection of HCSVs in a MF compared to the injection without the MF. The therapeutic efficacy of HCSVs was raised with the aid of an exogenous MF. The average tumor weight of the TRAMP-C1-bearing mice treated with HCSVs reduced from 1.4 ± 0.3 g to 0.4 ± 0.3 g compared to 3.1 ± 0.2 g in the PBS-treated control group; the percentage of mature CD80^+^CD86^+^ DCs in lymph nodes increased from 27.9 ± 0.9% to 35.88 ± 1.8%; the proportion of infiltrated CD8^+^ T cells increased from 4.3 ± 0.5% to 7.7 ± 0.8% in the tumor area, and from 43.4 ± 0.9% to 46.2 ± 1.9% in lymph nodes (**Figure 9**) 181.

Overall, the magnetic field can manipulate ferrous metal agents and it may induce a magnetocaloric effect on some nanostructures. In this context, magnetic-driven forces and magnetic-induced temperature rises may be taken into consideration in the preparation of magnetic field-activated nanotheranostic agents.

#### 3.2.6. Combined stimuli-activatable theranostic nanomedicine

In this section, we briefly present two typical combined activatable strategies: sequential activation by combined endogenous stimuli and combined activation by endogenous and exogenous stimuli.

**Combined endogenous stimuli-mediated sequential activation.** Based on the distinct location of endogenous stimuli, a stepwise dual/multiplexed activation strategy by endogenous stimuli has been explored. In a common paradigm, extracellular enzymes or tumor acidosis help induce charge reversal or size reduction of the nanomedicine to achieve its high cellular internalization and deep tumoral penetration, while the intracellular elevated level of redox or enzymes could trigger robust drug release or aggregation 182-184.

**Figure 10:**
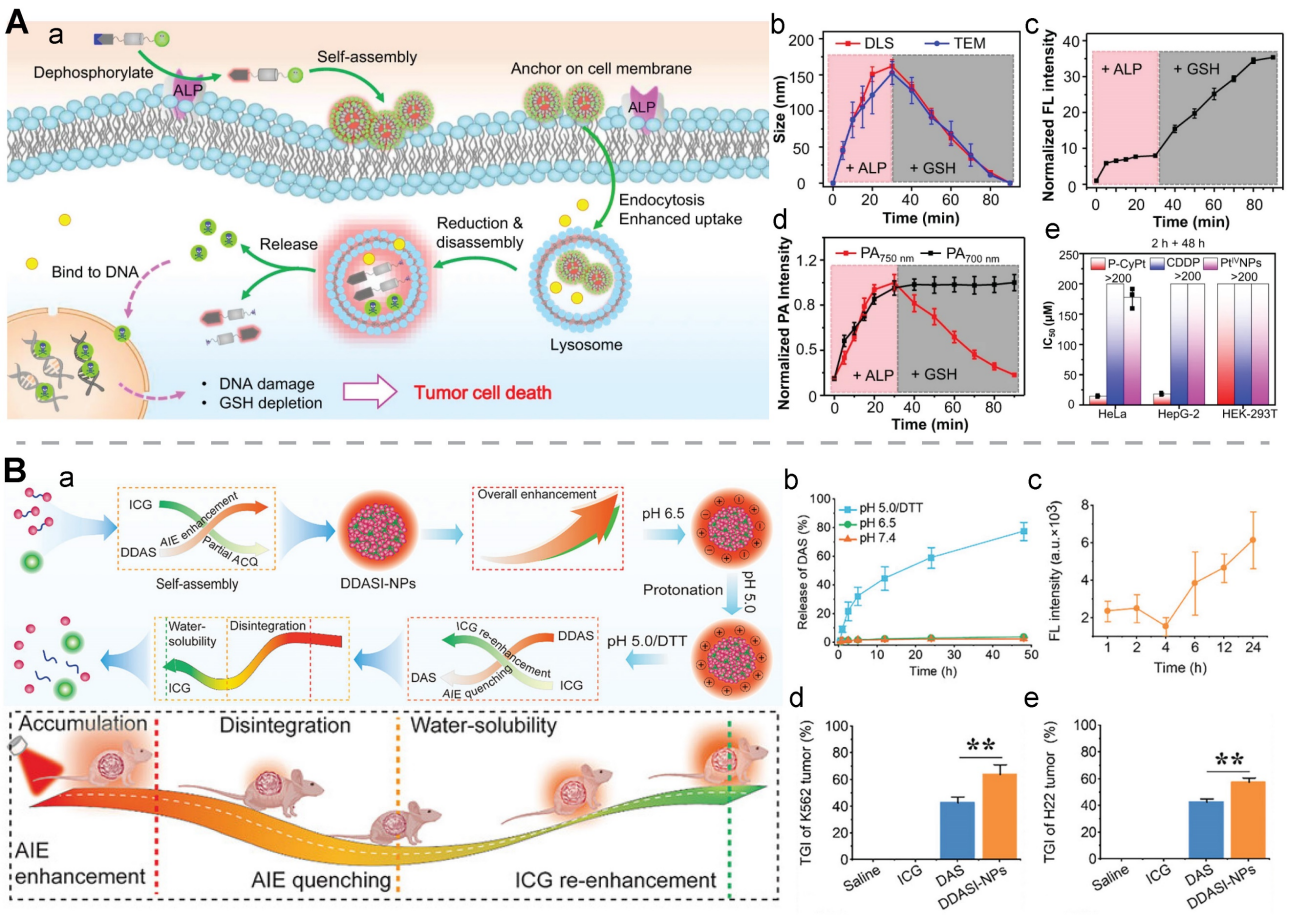
Dual endogenous stimuli-activatable nanomedicine for cancer theranostics. A) An enzyme/GSH-activatable fluorogenic cisplatin prodrug (P-CyPt) with extracellular self-assembly and intracellular disassembly for FL/PA imaging-guided chemotherapy of liver tumors. a) Schematic illustration of self-assembly and disassembly of the prodrug at different locations. b) Size, c) FL intensity, and d) PA intensity of this fluorogenic prodrug after sequential addition of alkaline phosphatase (ALP) and GSH. e) The IC_50_ values of the prodrug and its controls on tumor cells (HeLa and HepG-2) and normal cell (HEK-293T). Reproduced with permission 38. Copyright 2021 the Authors, published by Springer Nature. B) A pH/GSH-activatable theranostic nanoprobe (DDASI-NPs) for FL imaging-mediated spatiotemporal monitoring of the chemotherapeutic outcome. a) Scheme of the proposed working principle of the nanoprobe. b) Release profile of DAS at different conditions. c) Semiquantitative analysis of in vivo fluorescence intensity in tumor sites injected with this theranostic nanoprobe. Tumor growth inhibition (TGI) in d) the K562 tumor-bearing mice and e) the H22 tumor-bearing mice after different treatments. Reproduced with permission 185. Copyright 2023, Wiley-VCH.

**Enzyme plus redox.** In one study, alkaline phosphatase (ALP) on tumor cell membranes was harnessed to induce dephosphorylation and self-assembly of an activatable fluorogenic small-molecule Pt(IV) prodrug, P-CyPt, for enhancing its cellular uptake; the dephosphorylation procedure resulted in a turn-on of the FL signal (710 nm) and the PA signal (700 nm); self-assembly of the prodrug enabled a turn-on of the PA signal at 750 nm. Furthermore, the intracellular GSH triggered the disassembly of the prodrug to release cisplatin and diminish the PA signal (750 nm). This dual stimuli-activatable strategy offered spatiotemporal tumor delineation and drug-release monitoring, and it could hold great promise for precise cancer chemotheranostics (**Figure 10A**) 38. In another study, apurinic/apyrimidinic endonuclease 1(APE1), an intracellular enzyme upregulated by an elevated redox level during ferroptosis, was employed as the second stimulus to a nanomedicine after GSH exposure. A GSH/APE1 cascade-activatable nanoassembly was developed to realize real-time monitoring of APE1 dynamics and APE1-mediated drug release. In this nanoassembly, ultrasmall iron oxide nanoparticles were functionalized with DNA, which was labelled with Cy5.5 and contained GSH-sensitive disulfide linkers. DNA-functionalized magnetic nanoparticles and DOX self-assembled into a stable nanoassembly. In this design, GSH exposure enabled the first-stage disassembly, leading to a switch from T2 to T1 MRI; APE1 facilitated the second-stage disassembly with recovery of fluorescence signal and DOX release 186.

**pH plus redox.** It was reported that H^+^ facilitated dissociation of zeolitic imidazolate framework-8 coating and then GSH induced aggregation of the core gold nanorods via Au-thiol and zwitterionic electrostatic interaction. This design in the nanomedicine enabled a shift of the excitation laser wavelength for PAI and PTT from NIR-I (810 nm) to NIR-II (1048 nm) in a combined endogenous stimuli-activated manner 187. In another study, a pH/redox-responsive theranostic nanoprobe was developed with spatiotemporal dynamic fluorescence intensity conversion, and the nanoprobe was the self-assembly product of ICG and GSH-responsive dasatinib (DAS) dimers. In terms of imaging, the aggregation-induced emission (AIE) enhancement in tumor sites was achieved after accumulation of the theranostic nanoprobes, while intracellular pH/redox-induced disintegration of the nanoprobe led to the quenching of the AIE; the release of water-soluble ICG contributed to an enhancement in the fluorescence signal. This hypothesis was confirmed from in vivo fluorescence imaging of the K562-bearing mice: the FL intensity in the tumor tissues experienced its first drop at 4 h and then a steady increase in the following 20 h. For the therapeutic treatment, a high level of tumor accumulation of the nanomedicine and intracellular pH/redox-triggered DAS release contributed to a remarkable therapeutic efficacy compared to free DAS drugs on both K562 and H22 tumor-bearing mice (**Figure 10B**) 185. In another study, Fe(III)-coordinated croconaine molecules were mixed with bovine serum albumin (BSA) to fabricate the Cro-Fe@BSA nanomedicine that were sensitive to both pH and redox. The release rate of iron ions, the absorption rate of Cro-Fe@BSA, the generation rate of •OH radicals, the PA signal, and the r_1_/r_2_ ratio were improved as pH decreased. The reversible absorbance of the Cro-Fe complex indicated that GSH could facilitate the Fe^3+^-to-Fe^2+^ conversion. This pH/redox dual stimuli-activatable strategy promoted a synergistic antitumor effect of ferroptosis and photothermal therapy. CLSM imaging of BIDIPY-stained treated cells revealed that the green fluorescent intensity (an indicator of lipid peroxidation formation) in Cro-Fe@BSA-treated cells was much stronger than that in the cells treated by the control, Cro@BSA, and Cro@BSA/laser. Notably, the green fluorescent intensity of the Cro-Fe@BSA-treated cells increased by 1.67-fold upon exposure to light irradiation and decreased by 5.1-fold after adding a ferroptosis inhibitor. These findings together with regulated expression of GPX4 and HSP70 indicated that the generated photothermal effect aided in generation of •OH radicals and lipid peroxide. Moreover, the employment of Cro-Fe@BSA with the PA and MR imaging properties could help determine the optimal therapeutic window time (24 h) 188.

**Figure 11:**
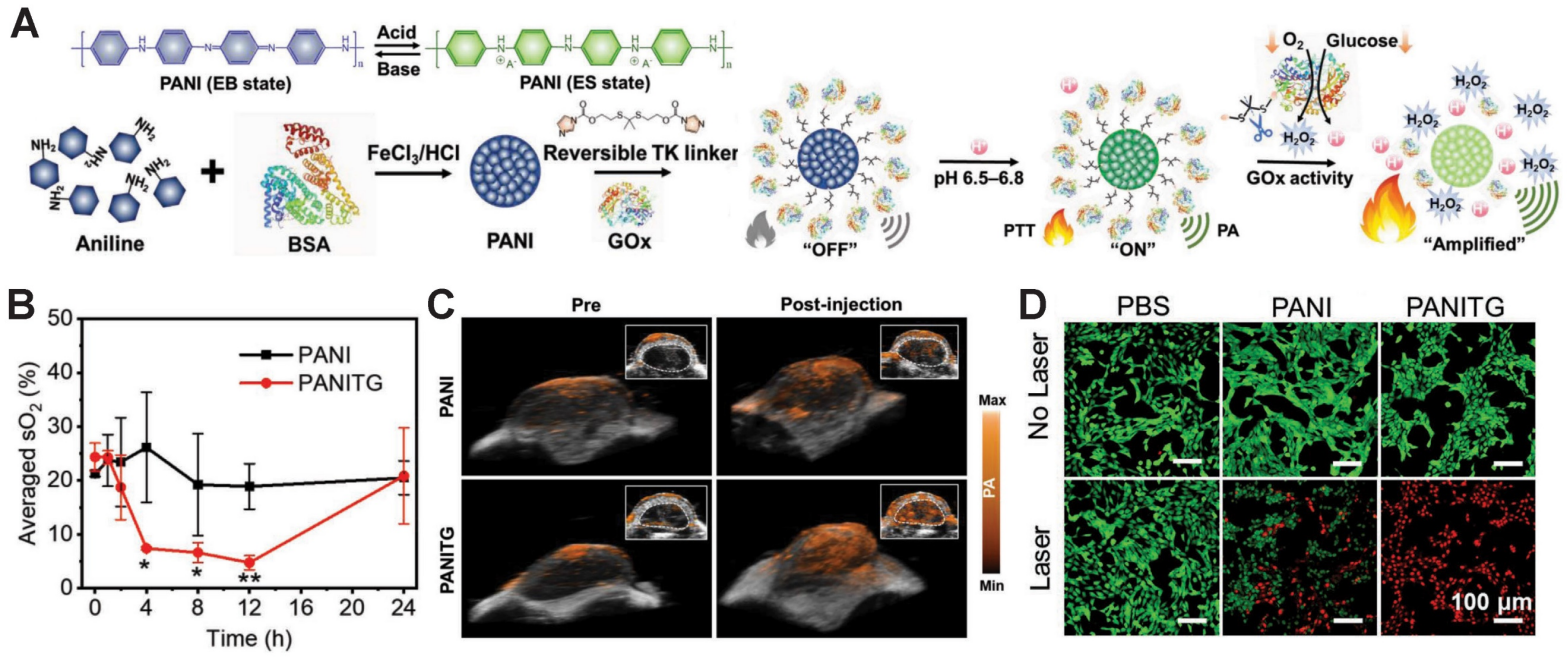
A pH/glucose/H_2_O_2_ triple-activatable conjugated polymeric nanoplatform (PANITG) for activatable photoacoustic imaging, photothermal therapy, and starvation therapy. A) Schematic of the activation mechanisms for imaging and therapy of PANITG. B) Quantification of the volume-averaged PA signal and the oxygen saturation (sO_2_) level at the tumor site and C) 3D PA images of tumor sites treated with PANI and PANITG. D) Live-dead cells staining of 4T1 cells after different treatments. Reproduced with permission 189. Copyright 2022, Wiley-VCH.

**pH plus redox plus glucose.** *In vivo* application of companion diagnostics was demonstrated with a glucose oxidase (GOx)-engineered polyaniline-based nanoplatform via a reversible TK linker (denoted as PANITGs). In this nanoplatform, pH-activatable polyaniline acted as a PTT agent and GOx in PANITGs reacted with glucose to generate H_2_O_2_ and glutamic acid. The wavelength of PANITGs redshifted under an acidic tumor environment. The PTT and PAI by the nanoplatform were amplified with a self-destructive effect induced by GOx catalysis-mediated breakage of a H_2_O_2_-clevable thioketal linker. The PA signal of the tumor area reached a peak at 4 h post-injection of the polyaniline (PANI) nanostructure and PANITG with an SNR of 2.0 and 2.9, respectively, indicating their ability to image the tumoral microenvironment. PA images with multi-wavelengths confirmed that the hemoglobin-oxygen saturation percentage distinctly declined from 24.4% to 7.4% in the PANITG-treated mice, comparing with a negligible change in the percentage in the mice treated with PANI, revealing the GOx-assisted starvation therapeutic role of PANITG (**Figure 11**) 189.

**Integrated endogenous stimuli with exogenous stimuli-mediated activation.** Integration of a remote exogenous stimulus with endogenous stimuli has been recently considered for designing activatable theranostic nanomedicine for more precise tumor control 190.

**Light + endogenous stimuli-induced activation.** In the light-induced theranostic nanomedicine, light-exerted physical stress, nanostructure-enhanced ROS generation, and/or a temperature rise could be the activation effects for the nanomedicine. Light in combination with endogenous stimuli would expand the avenue of intelligent nanomedicine for cancer theranositcs.

**Figure 12:**
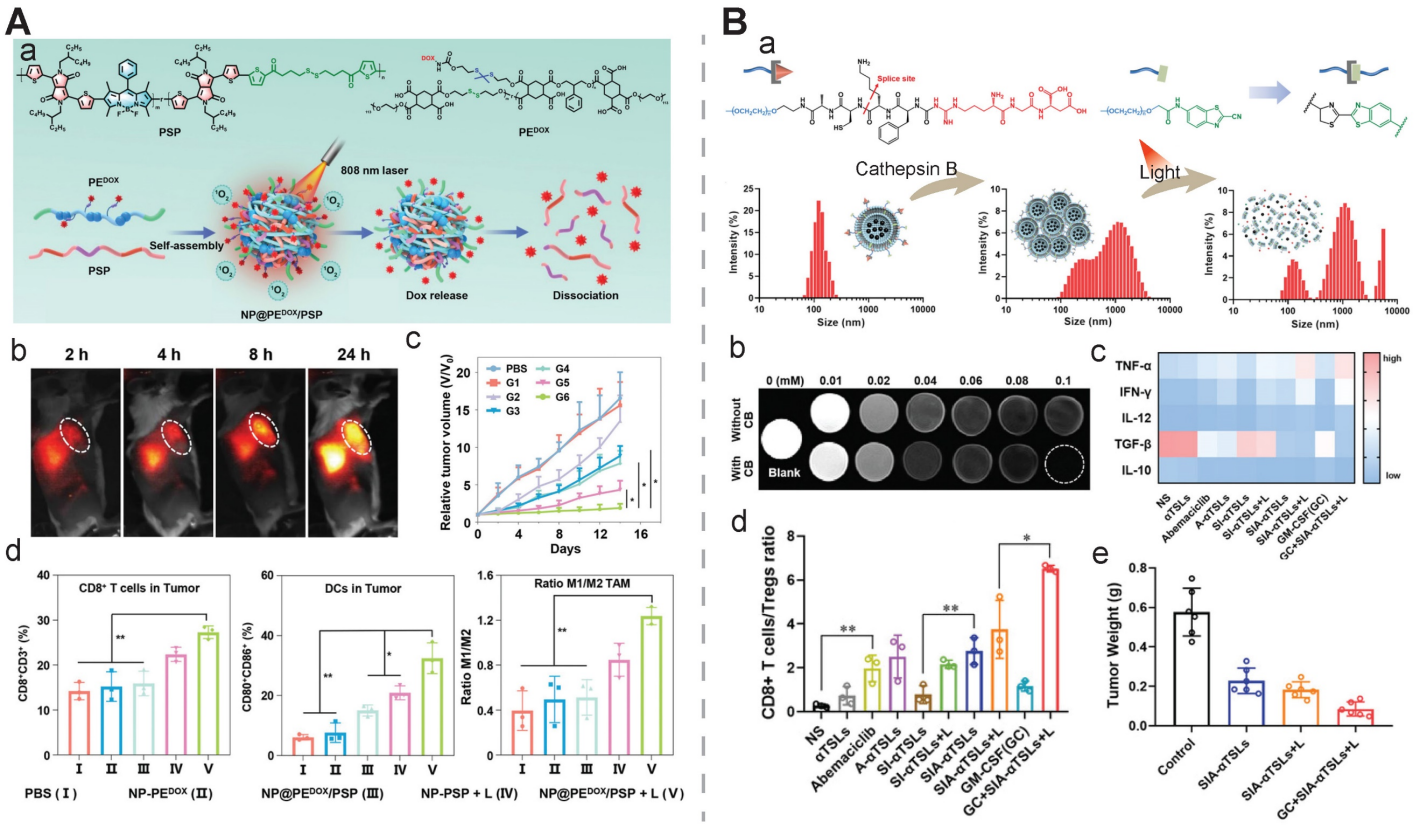
Combined exogenous/endogenous-activatable nanomedicine for cancer theranostics. A) Light/GSH-activatable pseudo-semiconducting polymeric nanoparticles (NP@PE^DOX^/PSP) for NIR-II fluorescence imaging, photodynamic immunotherapy, and photo-activated chemotherapy. a) Schematic of the preparation process of NP@PE^DOX^/PSP and its activation for imaging and therapy. b) *In vivo* NIR-II images of a murine tumor model treated with NP@PE^DOX^/PSP at different time points. c) Tumor growth curves of the mice groups treated with different methods, including PBS, NP-PSP (G1), DOX (G2), NP-PE^DOX^ (G3), NP@PE^DOX^/PSP (G4), NP-PSP + L (G5), and NP@PE^DOX^/PSP + L (G6). d) Quantitative analysis of CD8^+^ T cells in the tumor site and the spleen, CD80^+^CD86^+^ DCs in the tumors, and the M_1_/M_2_ ratio in the tumors from different groups. Reproduced with permission 191. Copyright 2022, Wiley-VCH. B) Light/enzyme-activatable SIA-αTSLs for MRI/NIRF-guided photothermal therapy. a) Changes in the chemical structure and hydrodynamic size via DLS of SIA-αTSLs after sequential activation by cathepsin B and light. b) T2WI of SIA-αTSLs incubated with/without cathepsin B. c) Intratumoral cytokines; d) ratios of CD8^+^ T cells to Tregs in the tumor tissue; and e) tumor weights at the endpoint of the CT26-bearing mice after different treatments. Reproduced with permission 196. Copyright 2022 the Authors, published by Wiley-VCH.

Light/redox activation. A self-sacrificed nanoassembly (NP@PE^DOX^/PSP) self-assembled from a pseudo-semiconducting polymer (PSP) with a photo-theranostic property and DOX-conjugated amphiphilic polyester (PE^DOX^). In this nanostructure, disulfide bonds were introduced in both PSP and PE^DOX^ for constructing biodegradable nanostructure and ROS-sensitive thioketal linkers at the DOX-conjugated site for realizing DOX release via ROS stimulation indirectly generated by light. HPLC analysis revealed that NP@PE^DOX^/PSP realized light-induced DOX release, while a negligible DOX amount was released without light irradiation. An increase in the elution time points of PSP and PE^DOX^ in the GPC chromatogram after incubation with GSH also supported the GSH-responsive degradability of this nanostructure. Notably, a continuous enhancement in the fluorescent intensity was seen at the tumor site in the first 24 h after *i.v.* injection of NP@PE^DOX^/PSP, indicating a time point of 24 h could be appropriate for subsequent light irradiation. The therapeutic outcome of NP@PE^DOX^/PSP through light-assisted combined therapy was confirmed with a significant tumor suppression effect and an enhancement in the percentage of infiltrated CD8^+^ T cells for up to 28.4% (**Figure 12A**) 191. Another study explored the manipulation of the aggregation status of a nanomedicine through the intracellular redox stimuli to trigger potent NIR-II light absorption for PAI and PTT. In this design, self-assembled gold nanochains were encapsulated in mesoporous silica nanoparticles (AuNCs@SiO_2_), and citrate ligands were coated on gold nanoparticles. The ligands were detached upon exposure to over-expressed H_2_O_2_ in the TME, contributing to a potent PCE of 82.2% via improved charge transfer between adjacent gold nanoparticles 192.

Light/pH activation. One recent study explored spatiotemporal control of metal-free tumor ferroptosis by a pH/light-activatable nanomedicine. This nanomedicine was prepared from phenothiazine-fused oxazine biotinylated nanoparticles (PTO-Biotin NPs), an oxazine-based photothermal molecular assembly. An acidic lysosomal environment activated the pH-responsive photothermal ingredient PTO2, and NIR light activation enhanced lysosomal dysfunction through remarkable Fenton reaction-promoted ferroptosis 193. In another study, the NIR light-generated thermal effect along with the tumor acidosis was employed to trigger intelligent release of chemotherapeutic DOX from PCN-DOX@PDA, a DOX-encapsulated and polydopamine (PDA)-coated PCN-600 nanoparticle. The chelated Fe ions in PCN-600 facilitated magnetic resonance imaging and the PDA coating shell enhanced the imaging performance with a 4-fold increase in the r_2_ value (from 8.23 to 32.84 mM^-1^ s^-1^) 194. A smart PDT/PTT complementary therapeutic strategy in response to light/pH/hypoxia-associated enzyme was proposed. In the extracellular environment of tumor cells, a low pH triggered the disassembly of BDP-Oxide NPs for the following fluorescence imaging-guided PDT. After these NPs deeply penetrated into tumor tissues, cytochrome P450 in these deep tumor sites featured with a low oxygen tension reduced BDP-Oxide into BDP, thus activating photoacoustic imaging-guided PTT. As a result, this smart theranostic nanomedicine contributed to a high tumor inhibition rate of 94.8% on the HepG2 tumor-bearing nude mice 195.

Light/Enzyme activation. Aggregation of a nanomedicinal structure triggered by tumor-specific enzymes or other endogenous stimuli could enhance the photoactivation therapeutic efficiency. In one study, a SIA-αTSLs nanomedicine was obtained by encapsulating photosensitizers IR780, chemotherapeutic drugs abemaciclib, and magnetic Fe_3_O_4_ NPs in ACKFRGD-peptide and 2-cyano-6-amino-benzothiazole (CABT)-co-modified liposomes. In the presence of cathepsin B at the tumor site, the ACKFRGD peptide was cleaved to expose the 1,2-thiolamino groups of the AC peptide fragment, which would react with CABT via click cycloaddition to form nanoaggregates for enhanced near-infrared fluorescence imaging and MRI. Under this enhanced imaging guidance, the tumor sites were irradiated with NIR light. Thermal-enhanced drug release and thermal-induced damage resulted in a high tumor inhibition rate of 79.81 ± 5.30% **(Figure 12B)** 196.

**Magnetic field + endogenous stimuli-induced activation.** One common theranostic application of a magnetic field is to stimulate magnetic nanoparticles for magnetic hyperthermia therapy and T2WI magnetic resonance imaging. One recent study adopted the pH-activatable charge-reversible organic coating strategy on surface modification of magnetic nanoparticles for inducing their intracellular aggregation. Two pH-responsive nanosystems, A-M5&M20 and A-M20&M20, were prepared by integrating M20@DPA/HA with negatively charged magnetic nanoparticles with different sizes (M5 and M20). According to the TEM images, heating curves, and MR images of various samples (M5, M20, M20@DPA/HA, A-M5&M20 or A-M20&M20), pH-activatable aggregation formation resulted in with a size-dependent magneto-thermal conversion efficiency: the aggregates from A-M20&M20 displayed a 20-fold increase in the conversion efficiency compared to individual nanoparticle M5, and a more than two-fold increase in the size and the r_2_ value compared to the single magnetic nanoparticle. After various treatments of the orthotopic 4T1-bearing mice under an AMF, the highest tumor inhibition rate of 83.8% was achieved in the A-M20&M20-treated group 197.

**Ultrasound + endogenous stimuli-induced activation.** Ultrasound often acts as an activation source for sonosensitizers and/or an initiator for the opening of cellular barriers, while various endogenous stimuli aid in disintegrating a nanomedicine to release therapeutic/imaging agents. In one recent study, an Mn-doped hollow MSN with a sonosensitizer Rose Bengal and a NO donor SNO was developed for ultrasound-triggered SDT and nitric oxide therapy. In this design, pH and GSH promoted Mn^2+^ release for an enhanced contrast of MRI: a ~3-fold increase in the r_1_/r_2_ value was seen after this nanomedicine was incubated in a TME-simulated buffer (pH 5.2, 5 mM GSH) compared to that in a normal environment-simulated one (pH 7.4). Meanwhile, an escalated increase in ROS generated by Rose Bengal and NO released by SNO contributed to the production of highly reactive ONOO^-^ for a synergistic antitumor effect; the average tumor size in the group treated with this combined therapy (MH-SNO@RB + US) was significantly reduced by ~82% 66.

Overall, these combined endogenous stimuli-activatable strategies can be generalized for designing theranostic nanomedicine with an outstanding imaging/therapeutic performance. The perturbance in the TME after the interventions of nanomedicine with exogenous stimuli could be systematically profiled and endogenous stimuli could be identified to align with the perturbance in the TME to realize synergistic imaging or therapeutic effects.

## 4. Reflection and future perspectives

After an in-depth dive into the working principles, designs, recent advances, and current clinical status (**Table 4**) of stimuli-activatable nanomedicine for cancer theranostics, we provide a few reflections into this topic: a) A better understanding of tumor biology is essential for the optimization of stimuli-activatable nanomedicine, such as the altered concentrations of tumor biomarkers at different cancer types/stages and after different interventions; b) Insufficient stimulus-responsiveness of a nanomedicine may originate from an inadequate dose of endogenous stimuli or a relative low intensity of external stimulation, a weak response of activatable ligands in the nanomedicine to endogenous or exogenous stimuli or a poor efficiency in breaking them, or excessive consumption of endogenous stimuli (e.g., GSH); c) Novel and efficient stimuli-activatable linkers and nanostructures for the nanomedicine have been explored, while there lacks a systematic investigation into their breakage efficiencies towards their corresponding stimuli using a generalized nanoplatform; d) An incorrect or incompatible fabrication method or an inappropriate synthetic route may lead to pre-cleavage of sensitive linkers or nanostructures in the nanomedicine; e) Exogenous stimuli can exert stimulation in both direct and indirect manners, which should be taken into consideration for the design of nanomedicine for cancer theranostics; f) The “all in one” strategy prevails in the nanomedicine design: functional agents are integrated into one single nanocarrier to realize multiple modalities of imaging/therapy or imaging-guided therapy, which could significantly deter the clinical translation of stimuli-activatable nanomedicine. Instead, one single agent with a well-established mechanism of action could be considered for integration into nanomedicine.

Based on these reflections, we propose the following future directions in the development of activatable nanomedicine for cancer theranostics:

i. The “activator-quencher” strategy for activatable theranostic nanoagents has been demonstrated with great potential in clinical application, and more pairing agents should be discovered for on-demand drug release or site-specific signal turn-on.

ii. Tumor enzyme-activatable strategy may become predominant in the nanomedicine for cancer theranostics since other endogenous stimuli may not be tumor-specific during the treatment of cancer patients with other accompanying diseases.

iii. In-situ synthesis of theranostic nanoagents with an aggregation-enhanced performance is encouraging. For instance, co-injection of CBT-NOTA-^68^Ga and CBT-NOTA-Ga led to their self-assembly into NPs after exposure to a high level of furin, and these NPs displayed enhanced microPET imaging contrast at a reduced dose of the imaging radioisotope 43. Similarly, the stimuli-triggered self-assembly of theranostic radioisotopes may escalate their therapeutic effects.

iv. To address insufficient stimuli-responsiveness, supply of extra endogenous stimulus agents via nanocarriers could be considered to improve the diagnosis/therapeutic efficiency of the corresponding activatable theranostic nanomedicine. In addition, activatable ligands that efficiently respond to stimuli and stimuli-cleavable chemistry should be explored.

v. With biological processes unveiled after therapeutic treatment, more specific or over-expressed stimuli can be exploited to construct an activatable nanomedicine to predict its therapeutic response, enable early stratification, and realize synergistic effects.

vi. Effective generalized masking shields of the nanomedicine with a stimuli-induced detachment behavior should be promoted as they can reduce the toxicity of therapeutic/imaging agents in the nanomedicine and the nanomedicine could gain an accelerated clinical translation. Specific nanoengineering of the masking shields in the nanomedicine could be advantageous for some unique, advanced cancer types.

vii. Nanorobotics that are driven by external stimuli or local stimuli could arrive at the target site with a high penetration ability to achieve great accumulation. Nanorobotic components could be integrated into the nanomedicine for cancer theranostics.

viii. Advanced tools, such as omics (radiomics, proteomics, and metabolomics) and artificial intelligence could be embraced to optimize the nanomedicine design based on large-scale data analysis 198, 199.

## 5. Conclusions

Nanoengineering the structure of medicine for cancer theranostics has thrived in the last two decades, and the stimuli-activatable strategy is one of the most promising engineering methods. The use of endogenous or exogenous stimuli for developing stimuli-activatable nanomedicine improves the specificity of on-site imaging or theranostics in addition to a high level of nanostructures-mediated tumor accumulation and mitigated toxicity to normal tissues. With the efforts of experts in multiple disciplines, simple but effective nanomedicine with a distinct mechanism of stimuli-activatable synergistic action will be translated into clinical application and benefit cancer patients.

## Figures and Tables

**Table 1:** Selected activatable ligands toward endogenous/exogenous stimuli

Stimulus	Activatable ligand and descriptions	Chemical structure or transition process	Ref
pH	Amide bond or bridge: cleavage	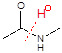	68
	Acetal bond: cleavage	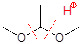	69
	Poly(*β*-amino esters): amine protonation	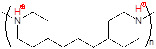	70
GSH	Disulfide bond: cleavage	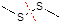	71
	Diselenide bond: cleavage	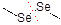	72
ROS	^1^O_2_-activatable thioketals: cleavage	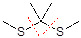	73
	H_2_O_2_-activatable ferrocene: hydrophobic to hydrophilic transformation	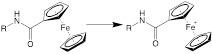	74
	H_2_O_2_-sensitive phenylboronic acid	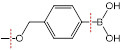	75
Enzyme	MMP2-cleavable peptide PLGIAG	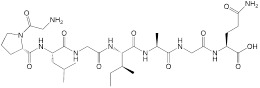	76
	Caspase 3/7-cleavage DEVD peptide (Asp-Glu-Val-Asp)	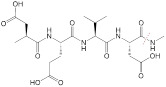	27
	Cathepsin B-cleavage GFLG tetrapeptide (Gly-Phe-Leu-Gly)	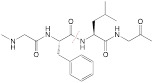	77
	GGT-cleavage γ-glutamyl moieties	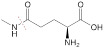	78
Hypoxia	A cleavable p-nitrobenzyl group by nitroreductase	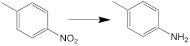	79
	Azobenzenes (AZO): reduction	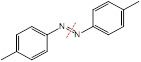	80
	2-nitroimidazole: hydrophobic to hydrophilic transformation	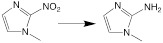	81
Light	Photocleavable linker (PCL)	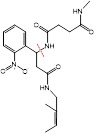	82
	Photolysis of *O*-nitrobenzyl ester	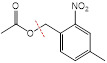	83
	Light-cleavable coumarin ester	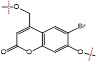	84
US	pMEMA: poly(methoxyethyl methacrylate)	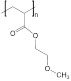	85
	Indirect breakage of thioketal bonds with the aid of a sonosensitizer	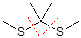	86
	Cleavage of ACVA C-N bonds	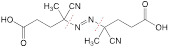	87
Ionizing radiation	Reduction of* N*-oxide	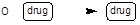	88
	Diselenid bond: cleavage	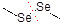	89
	Geometrical structure transformation: *cis*-GdAzo to *trans*-GdAzo	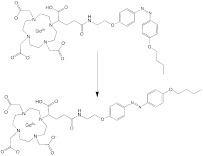	90
Thermal	Poly(N-isopropylacrylamide) (PNIPAAm)	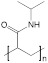	91
	Poly[(*N*-*N*-diethyl)acrylamide] (pDEA)	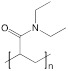	92
	Dipalmitoyl phosphatidylcholine (DPPC): gel to liquid-crystalline phase transition	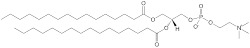	93

MMP2: matrix metalloproteinase 2; GGT: gamma-glutamyl transpeptidase; US: ultrasound; ACVA: 4,4'-Azobis(4-cyanovaleric acid).

**Table 2:** Selected examples of theranostic nanoagents

Nanoagents	Imaging methods	Therapeutic effects	Ref
MnO_2_ NPs	MRI	Hypoxia relief; immunomodulation; Fenton reaction	67
Gd-based NPs	MRI	Radio-sensitization; Gd-NCT	100
AuNPs	PAI; CT; SERS	PTT; radiotherapy	101
Iron oxide NPs	MRI; MPI	Ferroptosis; chemodynamic therapy; magnetic hyperthermia	102
Other heavy metal oxide-based NPs	CT	PTT; radiotherapy	103
Ag_2_S QDs	NIRF	PTT	104
Polydopamine	Thermal image; PAI	PTT	105
AIEgen	Photoluminescence imaging	PDT	106
Theranostic radio-isotopes-labeled NP	SPECT or PET	Radiotherapy	107

NPs: nanoparticles; Gd-NCT: gadolinium-neutron capture therapy; SERS: surface-enhanced Raman scattering; MPI: magnetic particle imaging; QDs: quantum dots; NIRF: near-infrared fluorescence; AIEgen: aggregation-induced emission luminogens; Theranostic radionuclides: ^47^Sc, ^64^Cu, ^67^Cu, ^67^Ga, ^77^As, ^111^In, ^117m^Sn, ^123^I.

**Table 3:** Representative examples of stimuli-activatable theranostic nanomedicine

Stimuli	Activatable theranostic nanomedicine	Imaging approaches	Therapeutic methods	Tumor models	Ref
pH	PPD@HPAP-CPDs/Rapa	FLI	Immunotherapy, chemotherapy	4T1 (breast cancer)	108
	AuNNR-DCNP Ve	NIRF, PAI	RT, chemotherapy	MCF-7 (breast cancer)	109
	Au/BP@MS	MRI	SDT	MCF-7 (breast cancer)	110
Redox	P-DOA NPs	PAI	SDT	B16F10 (melanoma)	111
	MMV-Au-CDs-DOX	FLI	Chemotherapy, CDT	4T1 (breast cancer)	112
	FDINs	PAI, FLI	PTT, chemotherapy	4T1 (breast cancer)	113
Enzyme	Fe-*^d^*HA	PAI	PTT, ferroptosis	4T1 (breast cancer)	114
	NRh-G-NPs	NIRF	PTT	U87MG (glioma)	115
	DQP/DMe NPs	FLI	Chemotherapy	A549 (lung cancer)	116
Other endogenous stimulus	AgNPs@GQDs-Gox	Fluorometry, FLI	Staving-like/metal ion/TA-induced apoptosis therapy	K299 (anaplastic large cell lymphoma)	117
	AzoCyS-N NPs	NIRF, PAI	PDT, PTT	HeLa (cervical cancer)	118
	GNPs@MRM/HAL	NIRF, PTI	PDT	4T1 (breast cancer)	119
Light	LPAR-siRNA	TI, PAI, CT	Gene therapy, PTT	Panc-1 (pancreatic cancer)	120
	BLIPO-I/D	NIRF	Chemotherapy, PDT, PTT	SW1990 (pancreatic cancer)	121
	AIBME@IR780-APM NPs	NIRF, MRI	PDT, TDT	4T1 (breast cancer)	122
Other exogenous stimuli	SCAN	Sonoafterglow luminescence	Immunotherapy, SDT	4T1 (breast cancer)	123
	PTX-NFGs	MRI	MF-triggered chemotherapy	LCC-6-WT (breast cancer)	124
	^89^Zr-TiO_2_-MnO_2_	PET, CLI	CRICT	CT26 (colorectal cancer)	125

FLI: fluorescence imaging; SDT: sonodynamic therapy; CDT: chemodynamic therapy; TDT: thermodynamic therapy; TI: thermal imaging; CLI: Cherenkov luminescence imaging; CRICT: Cherenkov radiation-induced cancer therapy; MF: magnetic field.

**Table 4:** Stimuli-activatable nanomedicine for cancer imaging/therapy in clinical trials or practices

Stimuli	Activatable nanomedicine	Descriptions	Indications	Clinical status
pH	ONM-100	Micelle covalently conjugated to indocyanine green for imaging-guided surgery	Solid tumor; peritoneal metastases; lung maliganancies	NCT03735680 (phase II, completed); NCT04950166 (phase II, recruiting); NCT05048082(phase II, completed)
	NC-6300	Polymer micelle with hydrazine-linked epirubicin	Advanced solid tumors or soft tissue sarcoma	NCT03168061 (phase I/II, unknown status)
	CRLX101	Cyclodextrin-containing polymer with glycine-linked camptothecin	Advanced non-small cell lung cancer	NCT01380769 (phase II, completed)
Redox	Mirvetuximab soravtansine	FRα-targeted antibody conjugated to maytansinoid DM4 via disulfide linker	Ovarian cancer	Approved, 2022
	Inotuzumab ozogamicin (Besponsa)	anti-CD22 conjugated to calicheamicin via disulfide and hydrazone linkers	Lymphoblastic leukemia	Approved, 2017
	LS301	A NIR fluorescent dye and an octapeptide that is cyclized through a disulfide bond	Breast cancer	NCT02807597 (phase I/II, recruiting)
Enzyme	Brentuximab vedotin (ADCETRIS®)	AntiCD30 conjugated to MMAE via cathepsin B-cleavable linkers	lymphoma	Approved, 2011/2012
	Paclitaxel poliglumex (Opaxio™)	Paclitaxel polymeric NPs sensitive to cathepsin B	Head and neck cancer; glioblastoma	Approved, 2012
	LiPlaCis	Liposomal formulated cisplatin sensitive to phospholipase A2	Advanced or refractory tumors	NCT01861496 (phase I/II, completed)
	CX-072	Anti-PD-L1 based on activatable Probody™	Solid tumor; lymphoma	NCT03013491 (Phase I/II, completed)
Hypoxia	18F-FMISO	Activatable PET probe	Anaplastic glioma	NCT01200134 (phase II, completed)
	[18F]HX4	Activatable PET probe	Head and neck and lung cancer	NCT02976883 (phase II, completed)
	^18^F-EF5	Activatable PET probe	Non-small cell lung cancer	NCT01017133 (phase I, completed)
Temperature	ThermoDox®	Thermosensitive liposome containing doxorubicin	Hepatocellular carcinoma	NCT02112656 (phase III, completed)
	CriPec® docetaxel	Thermosensitive polymeric micelles containing docetaxel	Solid tumors	NCT02442531(phase I, completed)
Radiation	NBTXR3	Hafnium oxide NPs for CT and radiosensitization	LA-HNSCC; Locally advanced soft tissue sarcoma	NCT04892173 (phase III, completed); European market approval, 2019
	AGuIX	Polysiloxane Gd-chelates based nanoparticles for MRI and radiosensitization	Multiple brain metastases	NCT03818386 (phase II, recruiting)
Magnetic field	NanoTherm®	Amino silane-coated Fe_2_O_3_ nanoparticles for MRI and MHT	Glioblastoma	Approved by EMA, 2010
	Magnetic nanoparticles	Magnetic thermoablation	Prostate cancer	Completed, NCT02033447 (early phase I, completed)
Light	AuroLase® (AuroShell)	PEG-coated silica gold nanoshells for NIR-activated thermoablation	Head and neck cancer	NCT00848042 (not applicable, completed)
	Photobac®	3-(1-Butyloxy)ethyl-3-deacetyl-bacteriopurpurin-18-n-butylimide methyl ester for intracavitary PDT	Glioblastoma or gliosarcoma	NCT05363826 (phase I, recruiting)
	Melanin	An endogenous light-absorber	Melanoma	NCT02613325 (phase I, completed)
Ultrasound	Sonazoid	Perfluorobutane gas-containing MB	Focal liver lesions	Approved (2007 in Japan and 2019 by NMPA of China)
	Microbubbles	US-triggered MB destruction for sonoporation	Colorectal cancer hepatic metastases	NCT03458975 (phase II, completed)
	Optison™	Perflutren protein-type A microspheres for US and US-triggered MB destruction	Hepatocellular Carcinoma	NCT03199274 (phase II, recruiting)

FRα: folate receptor α; MMAE: monomethyl auristatin E; FMISO: fluoromisonidazole; LA-HNSCC: locally advanced head and neck squamous cell carcinoma; EMA: European Medicines Agency; MHT: magnetic hyperthermia therapy; NIR: near-infrared light; MB: microbubble; NMPA: national medical products administration.

## Abbreviations

AIEgen: aggregation-induced emission luminogens
APC: antigen-presenting cell
ATP: adenosine-5'-triphosphate
BODIPY: boron dipyrromethene
CDT: chemodynamic therapy
CLI: Cherenkov luminescence imaging
CMC: chemistry, manufacture and control
CRICT: Cherenkov radiation-induced cancer therapy
CT: computed tomography
EPR: enhanced permeability and retention
FRET: fluorescence resonance energy transfer
GGT: gamma-glutamyl transpeptidase
GSH: glutathione
HIFU: high-intensity focused ultrasound
MF: magnetic field
MMP: matrix metalloproteinases
MRI: magnetic resonance imaging
MRgFUS: MR-guided focused ultrasound
PAI: photoacoustic imaging
PANI: polyaniline
PCE: photothermal conversion efficiency
PDMAEMA: poly (*N,N*-dimethylaminoethyl methacrylate)
PDT: photodynamic therapy
PET: positron emission tomography
PROTACs: PROteolysis Targeting Chimeras
PTT: photothermal therapy
QDs: quantum dots
ROS: reactive oxygen species
RT: radiation therapy
SCAN: sonoafterglow cancer nanoimmunotheranostic probe
SDT: sonodynamic therapy
SEC: size-exclusion chromatography
SI: signal intensity
SNR: signal-to-noise ratio
TADF: activate thermally activated fluorescence
TMEP: 2,2,6,6-tetramethylpiperidine
TEMPO: 2,2,6,6-tetramethylpiperidine 1-oxyl
TME: tumor microenvironment
TSLs: thermosensitive liposomes
US: ultrasound
USPIO: ultrasmall superparamagnetic iron oxide
